# Genome-wide identification and molecular evolution of *Dof* gene family in *Camellia oleifera*

**DOI:** 10.1186/s12864-024-10622-6

**Published:** 2024-07-18

**Authors:** Chun Fu, YuJie Xiao, Na Jiang, YaoJun Yang

**Affiliations:** 1 Key Laboratory of Sichuan Province for Bamboo Pests Control and Resource Development, Leshan Normal University, No. 778 Binhe Road, Shizhong District, Leshan, Sichuan 614000 China; 2 College of Life Science, Leshan Normal University, No. 778 Binhe Road, Shizhong District, Leshan, Sichuan 614000 China; 3 College of Tourism and Geographical Science, Leshan Normal University, No. 778 Binhe Road, Shizhong District, Leshan, Sichuan 614000 China

**Keywords:** *Camellia oleifera*, *Dof* gene family, Genome-wide identification, Molecular evolution, Collinearity analysis

## Abstract

DNA binding with one finger(*Dof*) gene family is a class of transcription factors which play an important role on plant growth and development. Genome-wide identification results indicated that there were 45 *Dof* genes(*ColDof*) in *C.oleifera* genome. All 45 ColDof proteins were non-transmembrane and non-secretory proteins. Phosphorylation site analysis showed that biological function of ColDof proteins were mainly realized by phosphorylation at serine (Ser) site. The secondary structure of 44 ColDof proteins was dominated by random coil, and only one ColDof protein was dominated by α-helix. *ColDof* genes’ promoter region contained a variety of cis-acting elements, including light responsive regulators, gibberellin responsive regulators, abscisic acid responsive regulators, auxin responsive regulators and drought induction responsive regulators. The SSR sites analysis showed that the proportion of single nucleotide repeats and the frequency of A/T in *ColDof* genes were the largest. Non-coding RNA analysis showed that 45 *ColDof* genes contained 232 miRNAs. Transcription factor binding sites of *ColDof* genes showed that *ColDof* genes had 5793 *ERF* binding sites, 4381 *Dof* binding sites, 2206 *MYB* binding sites, 3702 *BCR-BPC* binding sites. *ColDof9*,* ColDof39* and *ColDof44* were expected to have the most TFBSs. The collinearity analysis showed that there were 40 colinear locis between ColDof proteins and AtDof proteins. Phylogenetic analysis showed that *ColDof* gene family was most closely related to that of *Camellia sinensis* var. sinensis cv.Biyun and *Camellia lanceoleosa*. Protein-protein interaction analysis showed that ColDof34, ColDof20, ColDof28, ColDof35, ColDof42 and ColDof26 had the most protein interactions. The transcriptome analysis of *C. oleifera* seeds showed that 21 *ColDof* genes were involved in the growth and development process of *C. oleifera* seeds, and were expressed in 221 *C. oleifera* varieties. The results of qRT-PCR experiments treated with different concentrations NaCl and PEG6000 solutions indicated that *ColDof1*,* ColDof2*,* ColDof14* and *ColDof36* not only had significant molecular mechanisms for salt stress tolerance, but also significant molecular functions for drought stress tolerance in *C. oleifera.* The results of this study provide a reference for further understanding of the function of *ColDof* genes in *C.oleifera*.

**Supplementary Information:**

The online version contains supplementary material available at 10.1186/s12864-024-10622-6.

## Introduction

*Camellia oleifera*, a native plant of China, is distributed in Guangdong, Hong Kong, Guangxi, Hunan and Jiangxi, China [1, 2]. It is a perennial wild shrub of Camellia Linn. Because its seeds can be squeezed into oil (tea oil) for consumption, it is named “tea” [3]. Tea cake can be used as fertilizer, pesticide and feed; tea shell is an important raw material of activated carbon and tannin. *C.oleifera* can also be used for greening and beautifying the environment, or a good tree species for creating water and soil conservation forest, water conservation forest and biological fire prevention forest belt [4]. Studies have shown that some plants of the tea group of Camellia are rich in caffeine and other purine alkaloids and tea polyphenols, which have great economic value. China is the distribution center of Camellia plants, with rich resources [5]. Camellia plants mainly contain soap ridge, tannin and flavonoids, which have the effects of reducing blood sugar [6], weight loss [7], blood lipid regulation [8, 9], antioxidant [10], antibacterial [11, 12], anti-mutation [13] and so on. Among them, Camellia flower extract has been found to have certain whitening effect [14]. And the fermentation products of Camellia endophytic fungi are expected to be used as raw materials for medicines, cosmetics and health care products [15], which has high research and development value.

Transcription factors (TFs) are proteins that can specifically bind to specific sequences upstream of genes, thereby ensuring that target genes are expressed at specific strengths at specific times and locations [16]. As key regulatory proteins, TFs rarely function alone, and they usually recruit multiple TFs to achieve combined regulation of different metabolic pathways [17]. Dof (DNA-binding with one finger) protein belongs to a class of subproteins in the zinc finger protein family, which is a trans-acting factor of single zinc finger structure and a plant-specific transcription factor, playing an important role in the growth and development of plants. Because it has a unique single zinc finger conservative DNA binding domain rich in Cys residues, it is named Dof domain [18–20]. Dof domain is generally 200 ∼ 400 amino acids long and mainly consists of two regions: the conservative N-terminal and the variable C-terminal. The N-terminal contains a highly conserved Dof domain composed of 52 amino acids, in which the CX2CX21CX2C motif forms a single zinc finger structure. In this single zinc finger structure, 1 Zn2 + is covalently bound to 4 Cys residues. Zn2 + and Cys residues are the guarantee of Dof protein activity. The presence of bivalent ion chelators and any replacement of Cys residues will inactivate Dof protein. The Dof functional domain presents a C2C2 type zinc finger structure. Dof protein can not only bind to specific DNA sequences to regulate gene expression, but also interact with certain proteins to participate in the regulation of plant growth and development and abiotic stress response [21, 22]. The core sequence recognized by Dof protein is 5’- AAAG-3’ or 5’-CTTT-3’ [23]; its C-terminal amino acid sequence has a large variation and is the transcription regulatory domain of Dof protein [24], which can specifically recognize the cis-acting element with a sequence of 5’-AAAG-3’. Dof transcription factor plays an important regulatory role in the process of plant growth and development.

Dof transcription factors are involved in plant seed germination, tissue differentiation and widely involved in physiological and biochemical processes such as carbon and nitrogen metabolism [16, 17]. For example, in maize, the expression of *ZmDof36* can promote the biosynthesis of grain starch, while inhibiting the synthesis of soluble sugar and reducing sugar [25]. *Dof* transcription factors can not only respond to hormones and growth regulators, but also participate in light response. *Dof* transcription factors participate in defending cell-specific gene expression and participate in the process of plant morphological changes under adverse conditions [26, 27]. Studies have shown that *ZmDof1* can inhibit the expression of *Zm401*, thereby affecting pollen-specific expression [28]. *NtBBF1* (rolB domain B factor 1) in Dof transcription factors of tobacco regulates tissue-specific expression and auxin-induced expression [29] by interacting with the ACTTTA region of the promoter of oncogene rolB in its apical meristem and microtubule tissues, and thereby affecting root development [29]. *PbDof9.2* in pears can regulate flowering time. Overexpression of *PbDof9.2* in Arabidopsis can delay flowering time by interacting with the promoters of PbTFL1a and PbTFL1b [30].

In grain full stage of cereal crops, such as corn, rice, wheat, the identification of TGTAAAG sequences associated with grain full stage can regulate the biosynthesis of stored proteins and the expression of other proteins in grain full stage. Overexpression of *Dof* transcription factor *SRF1* in sweet potato significantly inhibited the transcription of *Ibβfruct2* gene. Thereby changing the carbon metabolism of sweet potato tubers and significantly reducing the accumulation of sucrose invertase have increased the starch content in sweet potato tubers by reducing the concentration of monosaccharides [31]. Kushwaha et al. [32] found that *ZmPBF* transcription factor of *Dof* family in maize could specifically bind to P-box, the cis-element in the promoter of *olysin* gene, activate the transcription of the gene, and affect the endosperm protein content. Wu [25] et al. found that *ZmDof36* in corn could positively regulate starch accumulation, and the expression of genes related to starch synthesis increased in overexpressed strains, and the starch content increased. In other species such as *Arabidopsis thaliana* [33], cotton [34] and *Chlamydomonas reinhardtii* [35], *Dof* family transcription factors have also been shown to increase lipid content in grain. In conclusion, transcription factors of *Dof* family play an important role in regulating seed storage protein and oil accumulation, and are the key to seed development.

The first *Dof* gene, *ZmDof1*, was discovered in maize [36], and the discovery of *Dof* genes in the green unicellular alga *Chlamydomonas reinhardtii*, the moss *Physcomitrella patens*, the fern *Selaginella moellendorffii*, and then extended to different taxa of vascular plants [37, 38]. At present, *Dof* TFs isolated from the whole genome of various plants are being discovered. Among the monocotyledonous plants, 30 rice [39], 30 sorghum [40], 119 sugarcane [41] and74 banana [42] were found. Among dicotyledonous plants, 114 cotton [43], 36 Arabidopsis [44], 33 peppers [45] and 34 tomatoes [46] were respectively found.

In recent years, the progress of plant genome sequencing has greatly promoted the identification of *Dof* genes in many plants [47]. However, the genome-wide structure and function of most *Dofs* remain to be elucidated, especially in *C. oleifera*, an important cash crop, and the identification and functional analysis of *Dof* transcription factors in *C. oleifera* genome are still blank. In this study, *Dof* transcription factors in *C. oleifera* genome were comprehensively identified, including chromosome localization, motif composition, gene structure, conserved domain, collinearity analysis and phylogenetic analysis. It is a foundation for the analysis of the potential role of *Dof* family members in the growth, development and tissue differentiation of *C. oleifera*.

## Materials and methods

### Data acquisition and identification of *Dof* gene family in *Camellia oleifera* genome

The genome sequences, protein sequences and gene annotation files of *Camellia oleifera* Abel. are downloaded in GitHub: (https://github.com/Hengfu-Yin/CON_genome_data) [48] or Zenodo: (https://zenodo.org/record/5768785). *C. oleifera* seed transcriptomics data was downloaded from NCBI(https://www.ncbi.nlm.nih.gov/geo/query/acc.cgi?acc = GSE190644) [48] The genome sequence, protein sequence, and gene annotation files for *Arabidopsis thaliana* L, *Camellia lanceoleosa* L, *Camellia sinensis* var. assamica cv. Yunkang 10 L, *Camellia sinensis* var. sinensis cv. Longjing 43 L, *Camellia sinensis* var. sinensis cv. Shuchazao L, *Camellia sinensis* var. sinensis cv.Tieguan Yin L and *Camellia sinensis* var. sinensis cv.Biyun L are available downloaded from National Genomics Data Center (https://ngdc.cncb.ac.cn/gwh/). The genome assembly accession numbers of the above research species in NGDC database (https://ngdc.cncb.ac.cn/gwh/) are as follows: GCA_904420315.1; GCA_025200525.1; GWHBQCE00000000; GWHAZTZ00000000.1; GWHBQCF00000000; GWHBQCG00000000; GWHBQCJ00000000. All specimen materials of *Camellia oleifera* Abel. and other research species are stored in the publicly available specimen database iPlant (https://www.iplant.cn/info/). The protein sequence and gene sequence of *Dof* gene family in *C. oleifera* and other species above were determined as *Dof* gene family by Pfam model (PF02701) of *Dof* gene family and SMART retrieval. The *Dof* gene family identified in the *C. oleifera* genome was named *ColDof1*-*ColDof45*, and TBtools were used to extract the protein sequence and genome sequence of their species [49]. All Dof protein sequences and gene sequences were used for subsequent bioinformatics analysis.

In order to study the effects of drought stress and salt tolerance on *C. oleifera*, we conducted experiments on the root system of *C. oleifera* treated with different concentrations of salt and PEG6000. PEG6000 is a response experiment that simulates drought stress. At Jun 15th to July 15th, 2023, we conducted different treatment experiments on the roots of *C. oleifera* under different conditions in Jiajiang County, Leshan City, Sichuan Province. The first experimental treatment was as follows: the treatment group treated one *C. oleifera* foot with NaCl solutions at concentrations of 5.0, 10.0, and 15.0 g/L for 72 h, while the control group had a concentration of 0 g/L. The second experimental treatment is as follows: the treatment group is one *C. oleifera* foot at PEG6000 of 3%, 6%, 9% concentration for 72 h. while the control group had PEG6000 of 0% concentration. All young leaf samples of *C. oleifera* under different conditions were stored in liquid nitrogen before being transported back to the laboratory for storage in -80 °C freezer for DNA and RNA extraction. The total RNA was extracted from young leaves of the treatment group and the control group. Reverse transcription of purified RNA into cDNA using a reverse transcription kit, and reverse transcribed cDNA was used for qRT-PCR to verify the expression of *Dof* transcription factor family genes in *C. oleifera*.

### Chromosomal location and gene structure analysis of *ColDof* genes

TBtools software was used to simplify and analyze the chromosomal location and gene structure of *ColDof* genes. TBtools software was used for chromosome localization and gene structure analysis maps.

### Physical and chemical properties analysis of ColDof proteins

Protparam (http://web.expasy.org/protparam/) online tools was used to analyze the molecular weight of protein, isoelectric point and instability index, the total average hydrophobicity, liposoluble coefficient of ColDof proteins.

### Subcellular localization and signal peptide analysis of ColDof proteins

CSBIO online website (http://www.csbio.sjtu.edu.cn/bioinf/Cell-PLoc-2/) and SignalP-4.1 online server (https://services.healthtech.dtu.dk/service.php?SignalP-4.1) were used to predict the subcellular localization and signal peptide of ColDof proteins, respectively.

### Transmembrane structure, hydrophilicity and phosphorylation site analysis of ColDof proteins

TMHMM server v. 2.0 (https://services.healthtech.dtu.dk/service.php?TMHMM-2.0) was used to analyze the transmembrane structure of ColDof proteins. ProtScale (https://web.expasy.org/protscale/) was used to analyze the hydrophilicity of ColDof proteins. NetPhos3.1 server (https://services.healthtech.dtu.dk/services/NetPhos-3.1/) was used to analysis phosphorylation sites of ColDof proteins.

### Secondary structure and tertiary structure analysis of ColDof proteins

The secondary structure of ColDof proteins was predicted by using SOPMA (https://npsa-prabi.ibcp.fr/cgi-bin/npsa_automat.pl?page=npsa_sopma.html). The SWISS MODEL software (https://swissmodel.expasy.org/) was used to predict the tertiary structure of ColDof proteins.

### Conserved motif analysis of ColDof proteins

MEME (http://meme-suite.org/tools/meme) was used to analyze the conserved motif of ColDof proteins. The motif number of parameters was set to 10, and all other parameters were default settings.

### SSR loci and microRNA prediction of *ColDof* genes’ promoter

TBtools software was used to process the *ColDof* gene family sequences, and then the online tool IPK (https://webblast.ipk-gatersleben.de/misa/) was used to predict the SSR locus in the promoter of *ColDof* genes. psRNATarget (https://www.zhaolab.org/psRNATarget) online tool was used to predict the miRNAs of *ColDof* genes.

### Cis-acting elements and transcription factor binding site analysis of *ColDof* genes

The 2000 bp upstream sequence of *ColDof* genes was extracted from *C.oleifera* genome sequences by using TBtools software based on the GFF3 file. PlantCARE online search tool (http://bioinformatics.psb.ugent.be/webtools/plantcare/html/) was used to predict the cis-elements that may be involved in the regulation of *ColDof* gene expression in *C. oleifera*. The 2000 bp upstream of *ColDof* genes were used to analyze transcription factor binding sites of *ColDof* genes by PlantRegMap (http://plantregmap.gao-lab.org/binding_site_prediction.php) online tool and TBtools software.

### Codon preference analysis of *ColDof* gene family in *C.oleifera*

CodonW tool was used to analyze codon preference of *ColDof* gene family, and PR2.plot was used to analyze codon preference of *ColDof* gene family.

### Collinearity analysis of *ColDof* gene family

The Fasta Stats tool in TBtools software was used to process the genome sequence and obtain the chromosome length file. Then One Step McScan-super Fast tool was used to compare *C.oleifera* protein itself, and blast results were obtained. Parse was also used to obtain the location of all *ColDof* genes based on GFF3 gene location information, and Advanced Circos was used to visualize the data.

### Phylogenetic analysis of ColDof proteins

Clustal X was applied to sequence comparison of ColDof protein sequences of *C.oleifera*. The phylogenetic tree of ColDof proteins was constructed with the software MEGA7 and Neighbor-Joining method (NJ) was adopted. The verification parameter bootstrap was repeated for 1000 times, and other parameters were the default values. The evolutionary tree of *C. oleifera* and *Arabidopsis thaliana* was constructed with the same method. A phylogenetic tree of Dof proteins sequences of 9 species, including *C.oleifera*, *Arabidopsis thaliana*, *Camellia lanceoleosa*, *Camellia sinensis* var. assamica cv. Yunkang 10, *Camellia sinensis* var. sinensis cv. Longjing 43, *Camellia sinensis* var. sinensis cv. Shuchazao, *Camellia sinensis* var. sinensis cv.Tieguan Yin and *Camellia sinensis* ar. sinensis cv.Biyun, was constructed by Maxmumm Like-lihood (ML) method. The calibration parameter bootstrap was repeated 1000 times.

### Protein-protein interaction analysis of ColDof proteins

ColDof protein sequences were uploaded to the interaction database String (https://string-db.org/) to analyze the protein-protein interaction of ColDof protein family. Reference species of ColDof proteins was set to “*Arabidopsis thaliana*”, keep the remaining parameters set to default, store the results in TSV format, import the TSV file into Cytoscape 3.8.2, and analyze the network (Cytoscape → Tools → Network analyzer → Network analysis → Analyze network), save the network analysis results, and reflect the size of the Degree using node size and color. The larger the node, the greater the Degree value; The thickness of the edge was used to reflect the size of the Combine score. The thicker the edge, the larger the Combine score. The core target was selected to create a protein interaction network diagram.

### Gene expression analysis of *ColDof* genes under 221 *C.oleifera* seed transcriptome and different stress conditions by qRT-PCR experiment

The FPKM value of gene expression of 45 *ColDof* genes under 221 *C.oleifera* seed transcriptome was downloaded from NCBI (https://www.ncbi.nlm.nih.gov/geo/query/acc.cgi?acc=GSE190644) [48]. Utilizing the SRplot platform(http://bioinformatics.com.cn/plot_basic_cutted_cluster_heatmap_plot_223) [50] Create a heatmap and analyze the FPKM values of 45 *ColDof* gene expressions.

All young leaf samples of *C. oleifera* under different conditions were stored in liquid nitrogen before being transported back to the laboratory for storage in -80 °C freezer for DNA and RNA extraction. The total RNA was extracted from young leaves of the treatment group and the control group by using plant RNA Extraction Kit from Beijing Tiangen Biotech Co., Ltd. Using the reverse transcription kit purchased from Beijing Tiangen Biotech Co., Ltd., the extracted and purified mRNA samples were reverse transcribed into cDNA. With the cDNA as a template, the 18S rRNA gene as an internal reference gene, and the designed qRT-PCR primers as a guide, the cDNA was subjected to PCR amplification under different NaCl and PEG6000 treatment conditions to obtain the expression levels of each *ColDof* gene. The relative expression level of the target gene *ColDof* was calculated by using the expression level of the reference gene as a reference. TBtools was used to process and heat map the obtained relative expression level of *ColDof* genes. All *ColDof* gene primers designed by TBtools Batch q-RT-PCR primer design tool used in the qRT-PCR validation experiment in this study are shown in Supplementary Table 1. The qRT-PCR primers used in this study were synthesized by Sangon Biotech (Shanghai) Co., Ltd on commission.

## Results

### Chromosomal location and gene structure analysis of *ColDof* genes

**Fig. 1:**
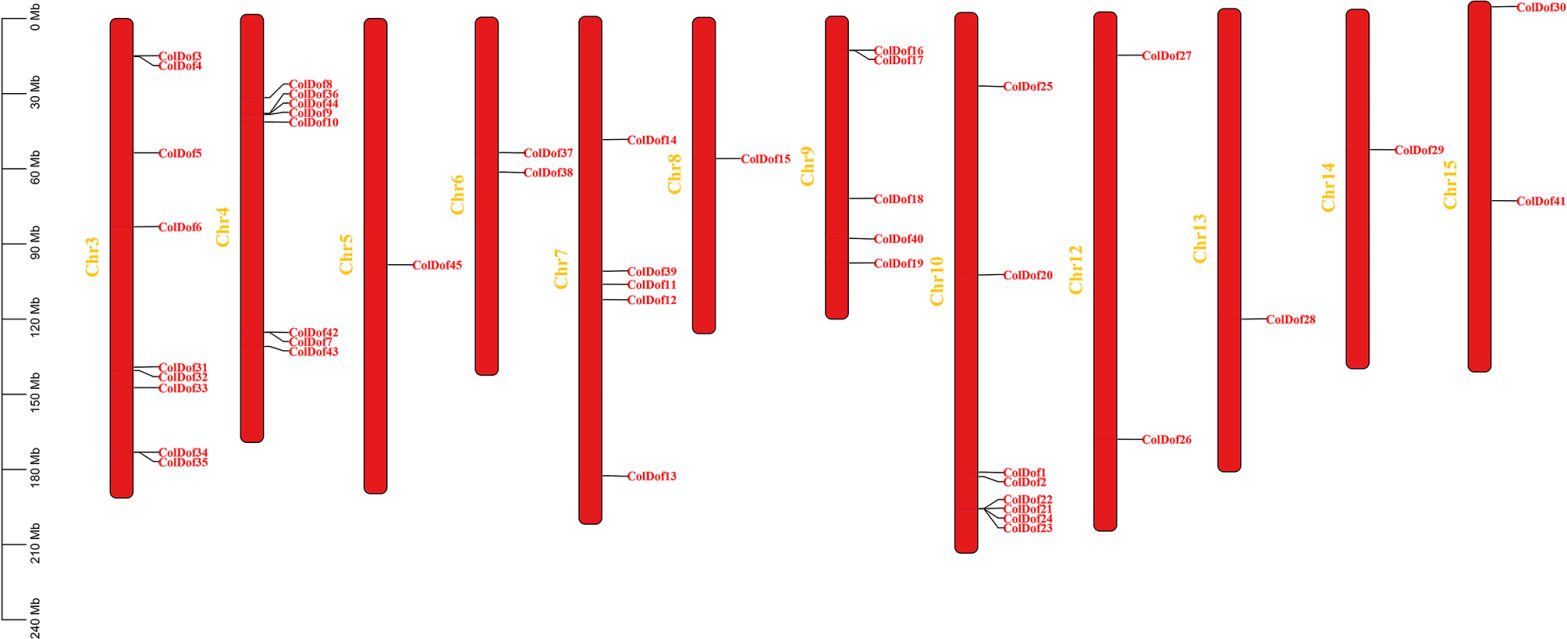
Chromosome mapping of *Dof* gene family in *C.oleifera*

45 *Dof* genes have been identified in *C. oleifera* genome. They were named *ColDoF1-ColDof45* according to their gene descriptions. *ColDof3* ∼ *ColDof6* and *ColDof31* ∼ *ColDof35* were located on chromosome 3 and had 9 genes. *ColDof7* ∼ *ColDof10* and *ColDof42* ∼ *ColDof44* were located on chromosome 4 and had 8 genes. *ColDof37 and ColDof38* were located on chromosome 6 and had two genes. *ColDof11* ∼ *ColDof14 and ColDof39* were located on chromosome 7 and had 5 genes. *ColDof16* ∼ *ColDof19 and ColDof40* were located on chromosome 9 and had 5 genes. *ColDof20* ∼ *ColDof25*,* ColDof1 and ColDof2* were located on chromosome 10 and had 8 genes. *ColDof26 and ColDof27* were located on chromosome 12 and had two genes. *ColDof30 and ColDof41* were located on chromosome 15 and had two genes. *ColDof45*,* ColDof15*,* ColDof28*,* and ColDof29* were located on chromosomes 5, 8, 13, and 14, respectively (Supplementary Tables 2 and Fig. 1).

**Fig. 2:**
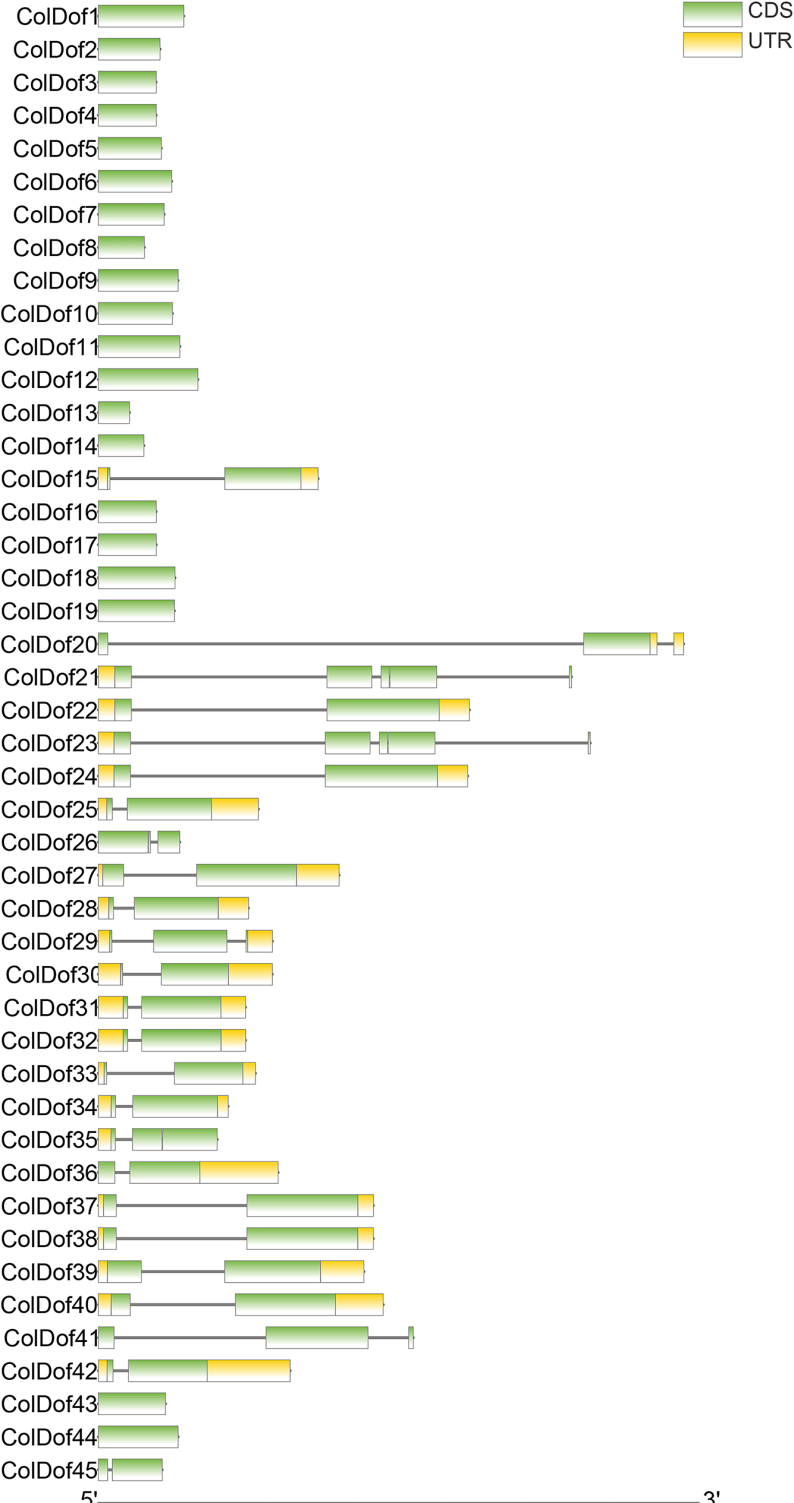
Gene structure of *Dof* family in *C.oleifera*

Among 45 *ColDof* genes, 22 *ColDof* genes were composed of introns and exons, among which *ColDof21 and ColDof23* contain one intron, and the other 20 contain two introns. Among 45 *ColDof* genes, the number of exons ranged from one to five, with 20 *ColDof* genes having one exon, 21 *ColDof* genes having two exons, two *ColDof* genes having three exons, and two *ColDof* genes having five exons. The family members with the most exons are *ColDof21* and *ColDof23* (Fig. 2).

### Physical and chemical properties of ColDof proteins

According to the physicochemical properties of ColDof proteins, the highest number of amino acids in ColDof proteins was ColDof25. The amino acid number of ColDof25 was 638 aa. The lowest number of amino acid in ColDof proteins was ColDof13. The amino acid number of ColDof13 was 120 aa. The overall amino acid content of ColDof proteins was between 120 aa and 638 aa. The average number of amino acids was 501 aa. The molecular weight of the protein ranges from 13.45278 to 70.04240 kD. The isoelectric point (pI) values ranged from 4.89 to 9.65, but 15 of the family members have theoretical isoelectric points less than 7, and the rest have theoretical isoelectric points greater than 7. The comparison of instability index values showed that the instability index values of ColDof06 and ColDof20 were lower than 40 and could be predicted to be stable proteins, while the instability index values of other sequences were all higher than 40 and were unstable proteins. The content of aliphatic index of ColDof ranged from 30.17 to 84.43, indicating that the thermal stability of this family of proteins varied greatly. Through the prediction of protein hydrophilicity/hydrophobicity, the GEAVY values of 45 ColDof were all negative, indicating that 45 ColDof members were hydrophilic protein members. The highest hydrophilic value was − 0.262, which was on ColDof12. The lowest hydrophilic value was − 1.191 in ColDof09. The total number of negatively charged residues in 15 of the 45 family members was greater than the total number of positively charged residues, indicating these 15 ColDof proteins were negative charge proteins. In 25 ColDof proteins, the total number of positively charged residues is greater than the total number of negatively charged residues, indicating these ColDof proteins were positive charge proteins. The total number of positively charged residues of the remaining five members was equal to the total number of negatively charged residues (Supplementary Table 3).

### Signal peptide and subcellular localization of ColDof proteins

According to the prediction of ColDof protein signal peptide, 45 ColDof proteins had no signal peptide, which suggested that they were all non-secreted proteins. Subcellular localization prediction of 45 ColDof proteins showed that all 45 ColDof proteins were located on the nucleus (Supplementary Table 3).

### Transmembrane structure, hydrophilicity and phosphorylation site of ColDof proteins

The transmembrane domain prediction analysis of ColDof proteins showed that none of 45 ColDof protein members had transmembrane phenomenon, so it was inferred that ColDof protein was non-transmembrane protein.

Hydrophilic/hydrophobic analysis showed that the maximum hydrophilic values of ColDof protein members ranged from 0.978 to 3.133, and the maximum hydrophilic values ranged from − 4.056 to -2.133. ColDof20 had a maximum value of 3.133, and ColDof39 had a minimum value of -4.056. According to the law that the lower the amino acid fraction, the stronger the hydrophilicity and the higher the fraction, the stronger the hydrophobicity, it can be seen that serine 27 on ColDof20 had the strongest hydrophobicity. Arginine, the 20th loci of ColDof39, had the strongest hydrophilic value, and as a whole, the hydrophilic value was more and more dense than the hydrophobic value. Therefore, the expression of ColDof protein was hydrophilic and it can be considered that ColDof protein was a hydrophilic protein (Supplementary Table 4).

The phosphorylation sites analysis of ColDof proteins showed that there were 2201 serine (Ser) phosphorylation sites, 763 threonine (Thr) phosphorylation sites and 211 tyrosine (Tyr) phosphorylation sites in 45 ColDof proteins. The serine (Ser) phosphorylation sites of ColDof34 were the largest, with 129. There were 15 members with the maximum value of 0.998 at the serine (Ser) site, and ColDof27 had the largest number of serine (Ser) phosphorylation sites (84). ColDof08 had a maximum value of 0.983 at the threonine (Thr) site, which was 170 threonine (Thr). ColDof10 had a maximum value of 0.978 at the tyrosine (Tyr) site, which was a serine (Ser) in the 211 position. The total number of phosphorylation sites of ColDof27 was 120. Member ColDof08 had the least phosphorylation sites (31). As the serine content is the highest in this family as a whole, we can infer that the protein functions mainly through phosphorylation at the serine (Ser) site (Table 1).

**Table 1:** Phosphorylation sites analysis of ColDof proteins

Protein name	Phosphorylation site			Serine (S)		Threonine (T)		Tyrosine (Y)	
	Serine(S)	Threonine(T)	Tyrosine(Y)	Position	Max	Position	Max	Position	Max
ColDof01	80	19	4	40	0.996	186	0.927	313	0.977
ColDof02	24	19	3	75	0.988	107	0.971	40	0.666
ColDof03	29	16	2	96	0.997	65	0.958	40	0.666
ColDof04	28	17	2	96	0.997	65	0.958	40	0.666
ColDof05	33	14	3	98	0.996	84	0.914	57	0.908
ColDof06	30	12	6	99	0.998	56	0.939	268	0.762
ColDof07	36	7	5	96	0.993	70	0.782	60	0.862
ColDof08	14	15	2	169	0.997	170	0.983	73	0.678
ColDof09	51	19	7	116	0.996	78	0.919	232	0.876
ColDof10	50	31	7	192	0.994	84	0.928	211	0.978
ColDof11	54	13	3	107	0.996	79	0.971	41	0.666
ColDof12	34	13	7	240	0.996	124	0.976	253	0.896
ColDof13	32	9	4	84	0.995	73	0.977	7	0.696
ColDof14	43	14	3	69	0.996	95	0.945	25	0.666
ColDof15	40	23	6	95	0.997	74	0.939	53	0.666
ColDof16	42	12	4	84	0.995	73	0.977	7	0.696
ColDof17	43	11	4	84	0.995	73	0.977	7	0.696
ColDof18	38	12	7	87	0.991	65	0.939	273	0.918
ColDof19	45	14	4	97	0.998	90	0.971	52	0.666
ColDof20	32	14	8	131	0.995	56	0.964	238	0.958
ColDof21	71	28	4	46	0.998	130	0.912	152	0.666
ColDof22	73	32	4	46	0.998	130	0.912	152	0.666
ColDof23	68	18	4	46	0.998	130	0.912	152	0.666
ColDof22	70	33	4	46	0.998	130	0.912	152	0.666
ColDof23	43	16	5	141	0.998	108	0.923	9	0.953
ColDof24	31	11	7	32	0.998	98	0.939	177	0.881
ColDof25	84	33	3	262	0.998	99	0.974	157	0.678
ColDof26	56	19	4	143	0.996	240	0.944	9	0.873
ColDof27	65	14	4	101	0.997	74	0.901	59	0.666
ColDof28	60	11	5	97	0.997	76	0.751	258	0.856
ColDof29	33	14	3	56	0.996	102	0.923	9	0.893
ColDof30	34	13	3	56	0.996	102	0.923	9	0.893
ColDof31	33	10	8	97	0.998	76	0.872	126	0.848
ColDof32	59	11	5	57	0.995	103	0.923	9	0.873
ColDof33	61	8	7	114	0.998	26	0.758	214	0.888
ColDof34	55	9	6	221	0.998	135	0.958	110	0.666
ColDof35	80	18	3	77	0.988	9	0.942	119	0.678
ColDof36	76	18	3	32	0.992	9	0.942	119	0.678
ColDof37	63	26	4	311	0.998	142	0.953	226	0.808
ColDof38	59	28	6	90	0.996	338	0.96	336	0.911
ColDof39	80	26	6	103	0.998	122	0.912	84	0.884
ColDof40	51	14	7	178	0.998	96	0.958	211	0.757
ColDof41	34	25	7	176	0.982	129	0.961	183	0.965
ColDof42	51	19	7	116	0.996	78	0.919	232	0.876
ColDof43	33	5	1	85	0.995	66	0.914	39	0.97
ColDof44	80	19	4	40	0.996	186	0.927	313	0.977
ColDof45	24	19	3	75	0.988	107	0.971	40	0.666

### Secondary and tertiary structure of ColDof proteins

**Fig. 3:**
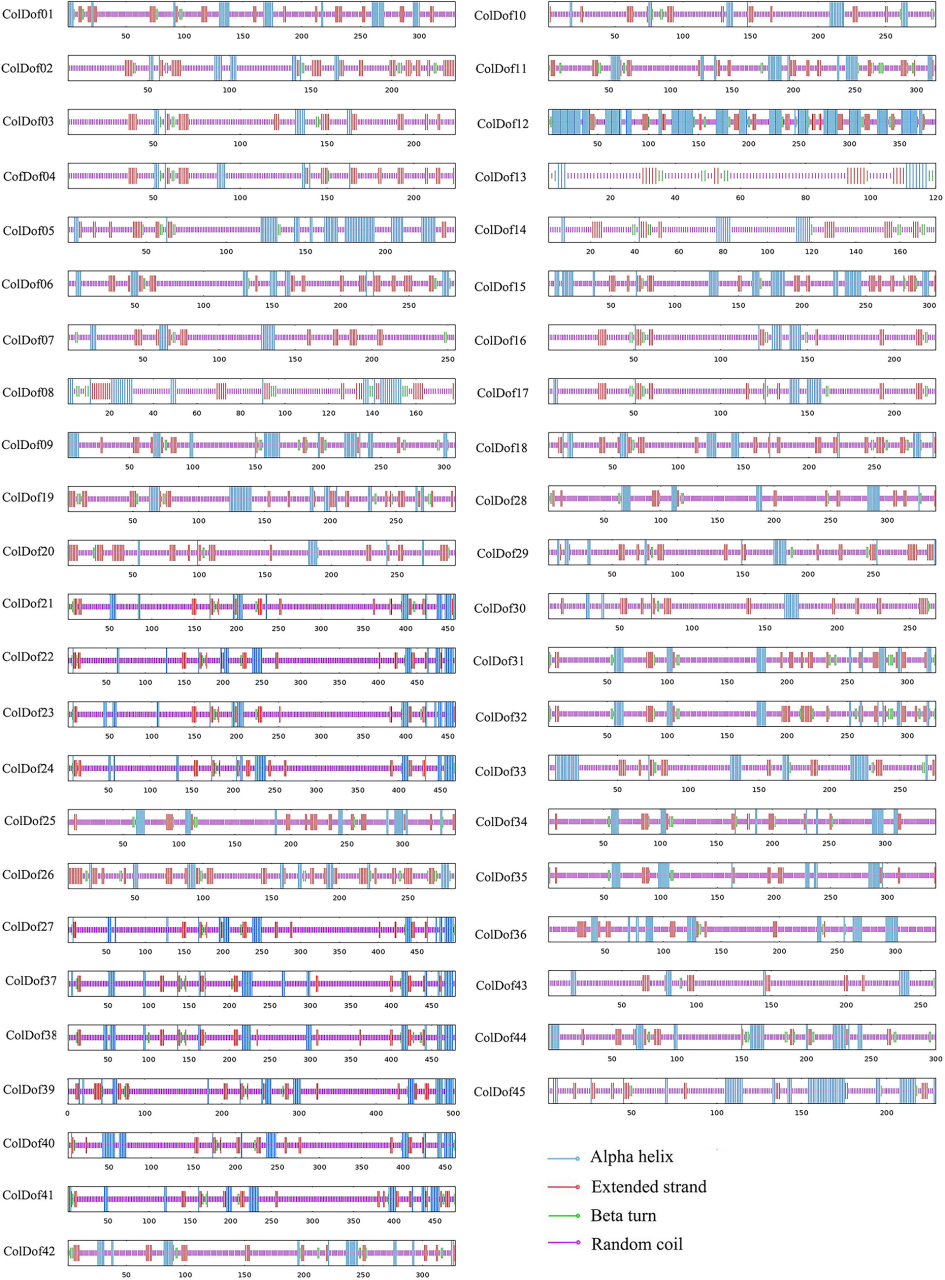
Secondary structure of Dof family in *C.oleifera*

From the prediction of secondary structure, it can be seen that 44 ColDof proteins were dominated by random coil, the proportion of which ranged from 57.87% (ColDof12) to 84.62%(ColDof43). Then, α-helix and extended chain structure accounted for 4.42% (ColDof20) ∼ 27.05% (ColDof05) and 7.19% (ColDof23) ∼ 23.51% (ColDof26), respectively, and β-turn accounted for the lowest proportion. It ranged from 1.10% (ColDof30) to 8.33% (ColDof13). The content of secondary structure of ColDof02, ColDof03, ColDof04, ColDof06, ColDof07, ColDof10, ColDof11, ColDof13, ColDof14, ColDof16, ColDof17, ColDof18, ColDof19, C olDof20, ColDof25, ColDof26, ColDof29, ColDof30, ColDof31, ColDof32 and ColDof43 were random coil > extended chain > α-helix > β-turn, that of ColDof42 was random coil > extended chain = α-helix > β-turn, and that of the other 22 ColDofs were random coil > α-helix > extended chain > β-turn. That of ColDof12 was mainly α-helix, and its proportion was 47.41%, which was α-helix > random coil > extended chain > β-turn (Supplementary Table 5, Fig. 3).

The tertiary structure of 45 ColDof proteins was predicted. According to the similarity of tertiary structure of each member, ColDof protein family could be divided into 40 categories. Among them, ColDof03 and ColDof04, ColDof16 and ColDof17, ColDof22 and ColDof24, ColDof37 and ColDof38 were the same category. And ColDof12 was different from the others in that its structure was mainly α-helix. From the tertiary structure diagram, it can be seen that the random coil in the Dof family members accounted for a large part, while other structures were scattered in the protein structure (Supplementary Fig. 1), which was consistent with the predicted results of the secondary structure.

### Conserved motif analysis of ColDof proteins

The online tool MEME was used to analyze 45 ColDof proteins’ sequences, 10 independent conserved motifs were identified. The 10 conserved motifs ranged in length from 15 to 50 amino acids. According to different Dof proteins have different number and types of motif, it is speculated that the reason may be because different Dof proteins play different functions in vivo. In 45 ColDof proteins, each ColDof protein contained at least 1 conserved motifs, 3 ColDof proteins contained 1 conserved motifs, and 4 ColDof proteins contained 10 conserved motifs. There are 32 ColDof proteins with 2 conserved motifs, 2 ColDof proteins with 5 conserved motifs, 1 ColDof protein with 6 conserved motifs, and 3 ColDof proteins with 7 conserved motifs. (Supplementary Figs. 2, 3)

### SSR loci analysis of *ColDof* genes’ promoter

The SSR loci analysis showed that SSR locis of *ColDof* genes were rich in repeat types such as mononucleotide, dinucleotide, trinucleotide and complex nucleotide. The number of each repeat type varied greatly, but single nucleotide repeats dominated, accounting for 42.65% of all SSRs with a total length of 462 bp. The distribution of single nucleotide SSR sites showed A clear preference, and the number of SSR sites for motif A/T was 25, while that for motif C/G was only 4. Dinucleotide repeats accounted for 20.59% of all SSRs, with a total length of 348 bp, and the main motif type was CT/TC. Trinucleotide repeats accounted for 29.41% of all SSRs, with a total length of 345 bp. Compound repeat sequences accounted for 7.35% of all SSRs, and the total length was 475 bp. Overall, the length of SSR locis in most *ColDof* genes in *C.oleifera* was less than 50 bp, accounting for 92.65% of all SSRs (Supplementary Table 6).

### MicroRNA prediction of *ColDof* genes in *C. Oleifera*

It was estimated that 232 miRNAs target 45 *ColDof* genes. The number of target genes of these mirnas was not very different, ranging from 1 to 23. Among them, ath-miR5658 had 14 target *ColDof* genes, ath-miR414 had up to 17 target *ColDof* genes, and ath-miR5021 had 23 target *ColDof* genes. And there was only one miRNA in 90 (ath-miR156c-3p, ath-miR156d-3p, ath-miR156f-3p, ath-miR161.2, etc.). The length of miRNA maturation sequence (5’-3’) was mainly 20 bp, accounting for 67.54% of all sequences. The mature sequence of miRNA with length of 19 bp accounted for 19.90% of all sequences, and the mature sequence of miRNA with length of 21 bp accounted for 8.81% of all sequences (Supplementary Table 7).

### Cis-acting elements analysis of *ColDof* genes

**Fig. 4:**
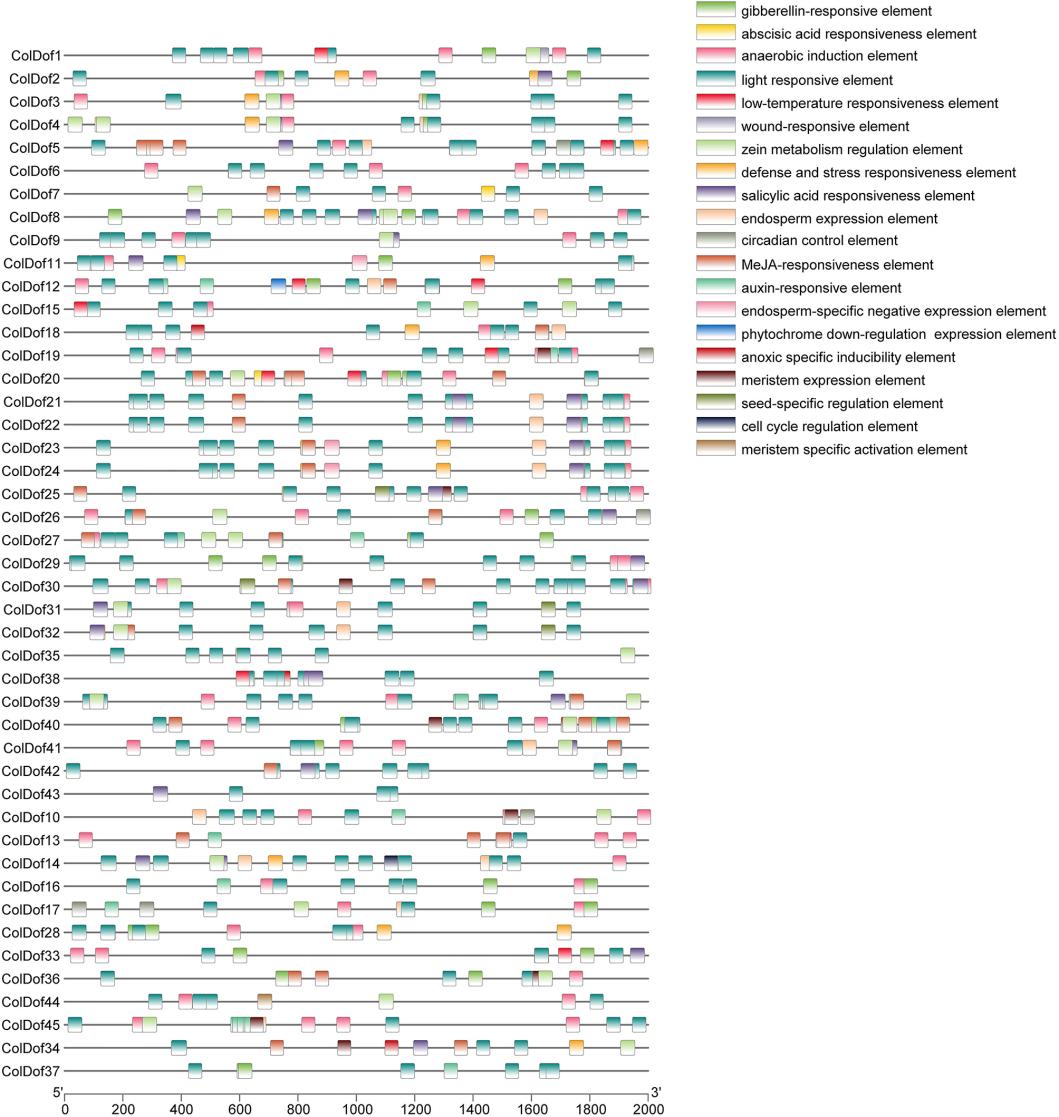
Cis-acting elements of *Dof* gene family in *C.oleifera*

By analyzing the 2000 bp upstream region of the promoter in *ColDof* gene family members, 20 cis-acting elements were screened. Photoresponsive elements were found in 45 *ColDof* genes, among which *ColDof21* and *ColDof22* promoter regions had the most photoresponsive elements (11). Abscisic acid response elements were found in 32 *ColDof* genes, among which *ColDof20* promoter region had the most abscisic acid response elements (6). MeJA response elements were found in 22 *ColDof* genes, among which *ColDof20* promoter region had the largest number of MeJA response elements (5). Anaerobic induction elements were found in 39 *ColDof* genes, among which *ColDof19* promoter region had the most anaerobic induction elements (5). Gibberellin response elements were found in 24 *ColDof* genes, among which the promoter region of *ColDof29* had the most gibberellin response elements (3). Salicylic acid response elements were found in 25 family members, among which *ColDof8*,* ColDof11*,* ColDof21*,* ColDof22* and *ColDof38* had the most salicylic acid response elements (2). Auxin response elements were found in 17 family members, and 5 auxin response elements in *ColDof45* promoter region were the highest. The metabolic elements of corn protein were found in 23 family members, among which *ColDof4* and *ColDof8* promoter regions were the most photoresponsive elements (3). Circadian control elements were found in 5 *ColDof* genes, among which *ColDof17* promoter region had the most circadian control elements (2). Seed-specific regulatory elements were found in four family members, and only one was found in the promoter region of *ColDof25*,* ColDof30*,* ColDof31* and *ColDof32*. Cold-responsive elements were found in 9 *ColDof* genes, among which ColDof12 and *ColDof20* had the most cold-responsive elements (2). The meristem expression element was found in 8 family members, and only one was found in the promoter region of *ColDof10*,* ColDof19*,* ColDof25*,* ColDof30*,* ColDof34*,* ColDof36*,* ColDof40* and *ColDof45*. Meristem specific activating element was found in *ColDof44.* A down-regulated expression element was found in *ColDof12*. Endosperm specific negative expression element was found in 3 family members, and only 1 element was found in the promoter region of *ColDof11*,* ColDof23* and *ColDof23*. Defense and stress response elements were found in 18 *ColDof* genes, among which *ColDof2*,* ColDof23*,* ColDof24* and *ColDof28* had the most defense and stress response elements (2). Endosperm expression elements were found in 17 *ColDof* genes, among which *ColDof4* and *ColDof14* promoter regions had the most expression elements (2). A cell cycle regulatory element was found in one family member, *ColDof14*. Hypoxia-specific induction elements were found in four *ColDof* genes, and only one was found in the promoter region of *ColDof18*,* ColDof34*,* ColDof38* and *ColDof40*. Only one *ColDof* gene, *ColDof1*, was found to be injury-sensitive. *ColDof* genes contain a variety of cis-elements and these family members are expected to play a key role in the response of oil tea to environmental stress and hormonal control (Fig. 4).

### Codon preference analysis of *ColDof* gene family in *C.oleifera*

The average content of the third codon is T3s > A3s > C3s > G3s. The average GC content (GC) of codons ranges from 0.34 to 0.58, with an average of 0.45. The GC of synonymous third codon bit (GC3s) ranges from 0.27 to 0.58, with an average of 0.38. Based on the analysis of codon related parameters of *Dof* gene family of *C.oleifera*. The content of GC and the mean value of GC3 are both less than 50%, indicating that AU is used more frequently than GC in the codon of the coding sequence of members of this family. Codon adaptation index (CAI) varied from 0.25 to 0.34, with an average value of 0.18.7, indicating that the Dof gene family had a low preference for codon selection. The optimal codon frequency (Fop) ranges from 0.33 to 0.50, with an average of 0.39. The codon bias index (CBI) ranges from − 0.18 to 0.17, with a mean of -0.03. The effective codon number (ENc) varied from 45.96 to 61.00, with a mean value of 51.54, indicating large differences among family members, relatively moderate expression levels, and low codon preference when encoding amino acids. The number of synonymous codons (L_sym) ranges from 115 to 2066, with an average of 587.11. The total number of amino acids (L_aa) ranged from 118 to 2132, with an average of 609.33. Protein Aromo ranges from 0.04 to 0.18, with an average of 0.10 (Supplementary Table 8).

There are 30 high-use codons (RSCU > 1), including 13 U terminals, 9 A terminals, 3 G terminals, and 5 C terminals (except stop codons UAA, UGA, and UAG, and start codons AUG and UGG). Of the 29 low-usage codons, 11 end in C, 10 end in G, 5 end in A, and 3 end in U. This indicates that the preference for high-usage codons ends at U and the preference for low-usage codons ends at C. In addition, the RSCU value of AGA is greater than 2, indicating a strong preference for this codon among members of *ColDof* gene family (Supplementary Table 9).

### Transcription factor binding sites of *ColDof* genes

**Fig. 5:**
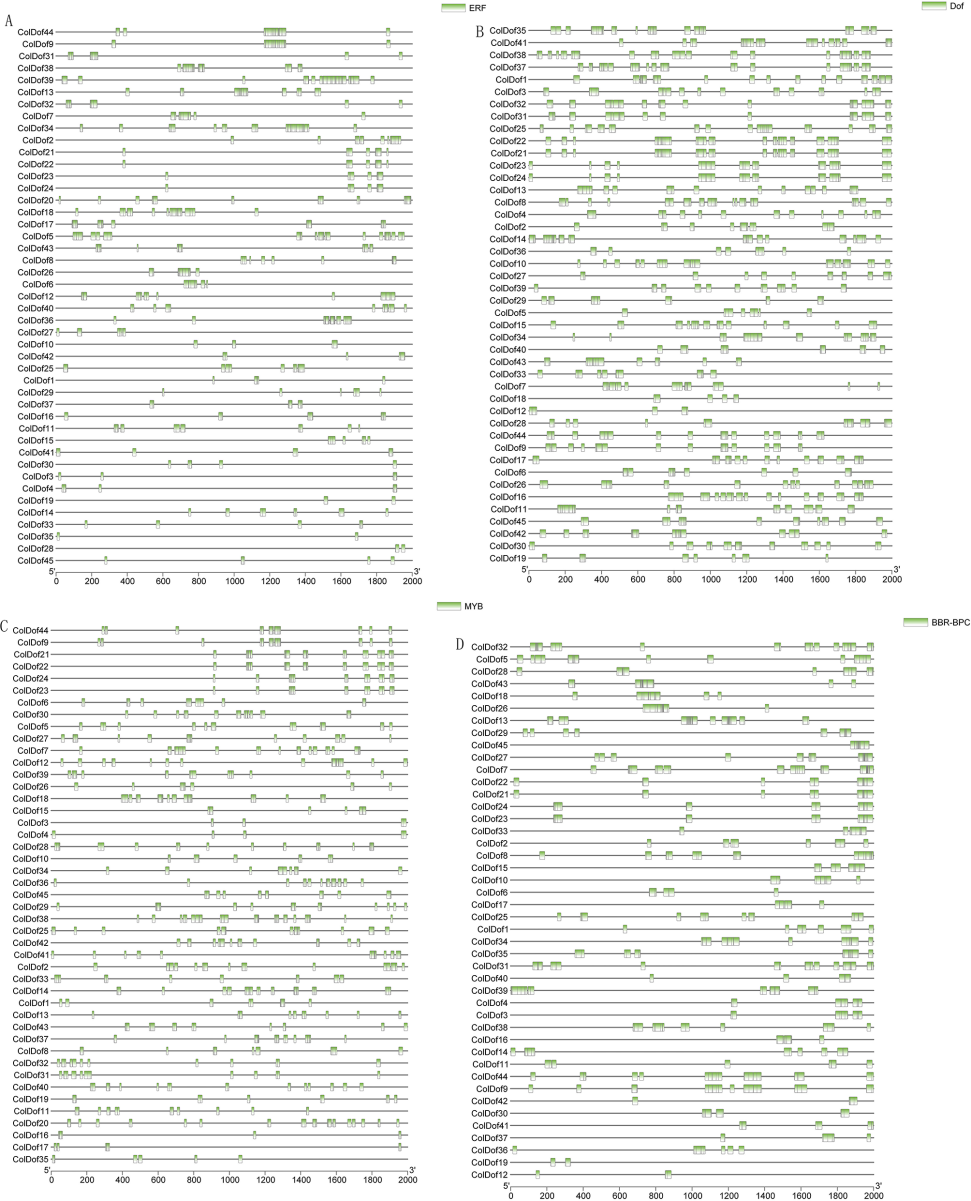
The ERF **(A)**, Dof **(B)**, MYB **(C)** and BBR-BRC **(D)** TF binding sites in the promoter region of the *ColDof* genes

Transcription factor binding site analysis showed that all *ColDof* promoter regions had dense TFBSs distribution. According to the number of binding sites, we selected three basic TFBSs for demonstration, among which ERF was the largest with 5793 (Fig. 5A), followed by Dof with 4381 (Fig. 5B), MYB with 2206 (Fig. 5C), and BCR-BPC with 3702 (Fig. 5D). Three ColDof (*ColDof9*,* ColDof39*,* and ColDof44*) are expected to have the most TFBSs. The prediction of TFBSs provides a basis for further identification and verification of target genes.

### Collinearity analysis of *ColDof* gene family

**Fig. 6:**
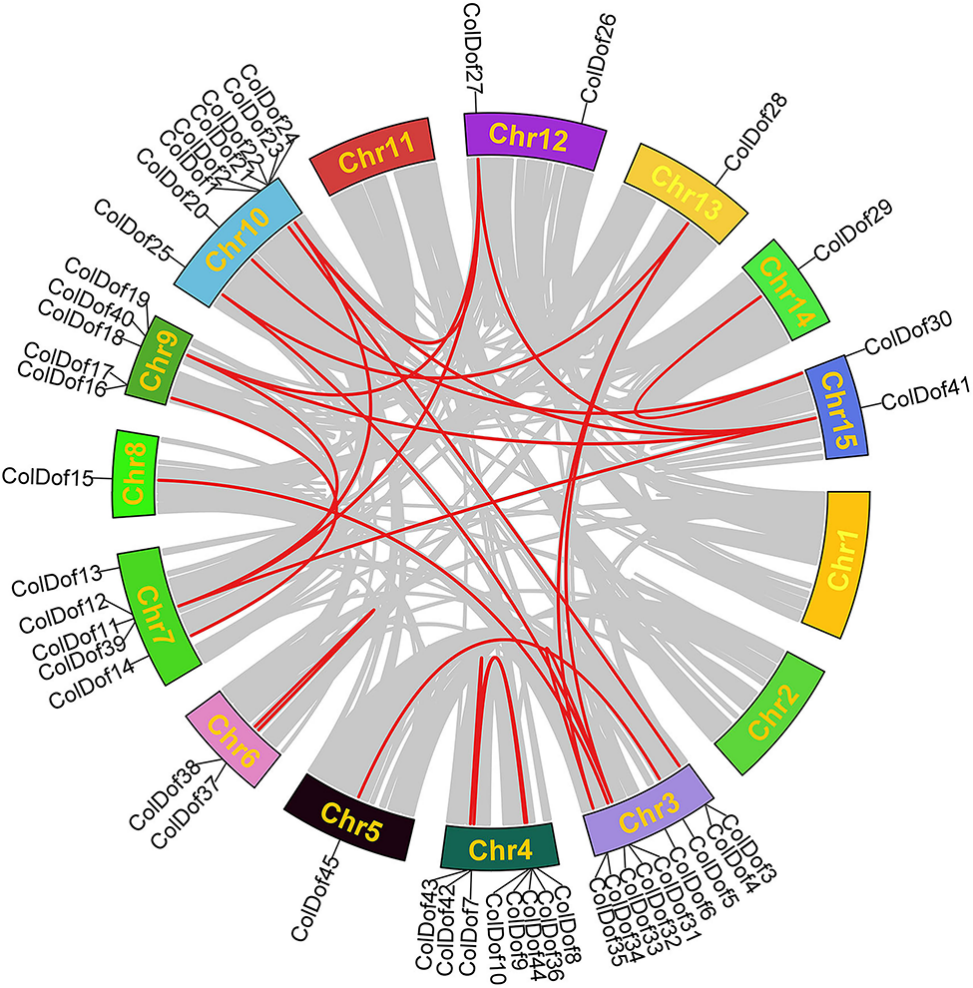
Collinearity analysis of *Dof* gene family in *C.oleifera*

Gene replication can occur in a variety of ways. In the process of biological evolution, gene families are mainly amplified by fragment replication, tandem replication and whole genome replication, and the replicated genes control the physiological and morphological evolution of plants. The association among gene family members was further studied by comparing ColDof proteins, and 24 pairs of fragment replicators were found in 45 *ColDof* genes (Fig. 6; Table 2). Of the 15 chromosomes, Chr3 and Chr10 have the most copies, with seven pairs, On Chr3, they are *ColDof2-ColDof3*,* ColDof5¬ColDof45*,* ColDof33-ColDof15*,* ColDof28-ColDof34*,* ColDof28-ColDof32*,* ColDof32-ColDof34*,* and ColDo f25-ColDof32*. On Chr10, they are *ColDof2-ColDof3*,* ColDof20-ColDof30*,* ColDof22-ColDof27*,* ColDof22-ColDof41*,* ColDof22-ColDof39*,* ColDof25-ColDof28*,* and ColD of25-ColDof32*. Chr12 has 4 pairs of replicant gene pairs, namely *ColDof27-ColDof41*,* ColDof27-ColDof39*,* ColDof27-ColDof40 and ColDof22-ColDof27*. There are at least one pair of replicators ColDof29-ColDof30 on Chr14. There are no *ColDof* gene replicators in Chr1, Chr2 and Chr11, and gene pairs in chromosomes exist in Chr3, Chr4 and Chr6. *ColDof* genes of *C.oleifera* were hypothesized to have undergone a certain scale of fragment replication events during evolutionary development (Fig. 6).

Since the Ka/Ks ratio is a good indicator of the selection pressure occurring at the protein level, we used TBtools software to estimate the Ks(synonymous) and Ka(non-synonymous) values as well as the Ka/Ks ratio. Ka/Ks < 1, Ka/Ks = 1 and Ka/Ks > 1 are generally considered to represent negative, neutral and positive selection, respectively. The Ka/Ks of 24 duplicate *ColDofs* were all < 1, ranging from 0.13 to 0.43, indicating that all duplicate gene pairs were under strong purification selection, which is consistent with the observation of other plants, such as apple and tomato (Table 2).

**Table 2:** Gene duplication types and Ka/Ks analysis for duplicated gene pairs of *ColDof* genes

Gene name	Gene name	Ka	Ks	Ka/Ks
*ColDof2*	*ColDof3*	0.31	0.82	0.38
*ColDof5*	*ColDof45*	0.36	0.84	0.43
*ColDof9*	*ColDof42*	0.63	NaN	NaN
*ColDof10*	*ColDof43*	0.21	0.51	0.40
*ColDof14*	*ColDof16*	0.14	0.76	0.19
*ColDof33*	*ColDof15*	0.12	0.39	0.31
*ColDof22*	*ColDof41*	0.28	1.56	0.18
*ColDof27*	*ColDof41*	0.23	0.62	0.37
*ColDof41*	*ColDof39*	0.34	1.23	0.27
*ColDof41*	*ColDof40*	0.35	1.33	0.26
*ColDof42*	*ColDof43*	0.64	2.52	0.26
*ColDof38*	*ColDof37*	0.55	2.23	0.25
*ColDof20*	*ColDof30*	0.24	0.69	0.35
*ColDof22*	*ColDof27*	0.16	0.72	0.22
*ColDof22*	*ColDof39*	0.25	0.61	0.41
*ColDof25*	*ColDof28*	0.23	1.33	0.17
*ColDof25*	*ColDof32*	0.15	1.02	0.15
*ColDof27*	*ColDof39*	0.31	1.45	0.21
*ColDof27*	*ColDof40*	0.18	0.79	0.23
*ColDof28*	*ColDof34*	0.34	1.92	0.18
*ColDof28*	*ColDof32*	0.41	NaN	NaN
*ColDof29*	*ColDof30*	0.11	0.84	0.13
*ColDof32*	*ColDof34*	0.19	1.18	0.16
*ColDof41*	*ColDof39*	0.38	NaN	NaN
*ColDof39*	*ColDof40*	0.27	0.89	0.31

In order to further investigate the evolutionary history of *Dof* genes in different species, we conducted a collinearity analysis of *Dof* gene families in *C.oleifera* and *A. thaliana*. The results showed that 40 pairs of *Dof* gene family were related to *C.oleifera*. Among them, the homologous gene pairs on Ol3 are the most, there are 9 pairs. They were *ColDof5-AtDof4*,* ColDof32-AtDof3*,* ColDof32-AtDof14*,* ColDof32-AtDof39*,* ColDof32-AtDof42*,* ColDof34-AtDof4*,* ColDof34-AtDof17*,* ColDof5-AtDof4*,* Coldof32-AtDoF17. ColDof34-AtDof14 and ColDof34-AtDof39*. Secondly, there were 4 homologous gene pairs on Ol4, Ol7, Ol9, Ol10, Ol13 and Ol15, and the least on Ol12 and Ol14, only 2 homologous gene pairs were *ColDof27-AtDof12* and ColDof27-AtDof5, respectively. *ColDof29-AtDof11* and *ColDof29-AtDof37* had no homologous pairs on Ol1, Ol2, Ol5, Ol6, and Ol11 (connected by pink lines in Supplementary Fig. 4A).

In order to study the evolutionary history of *Dof* gene in *C.oleifera*, we conducted a collinearity analysis of *Dof* gene family in *C.oleifera*. The results showed that 88 pairs of *Dof* gene family members were related to *C.oleifera*. It has the most homologous gene pairs on Ol3, with 16 pairs, They were *ColDof3-CalDof53*,* ColDof3-CalDof34*,* ColDof3-CalDof30*,* ColDof5-CalDof40*,* ColDof5-CalDof29*,* ColDof6-CalDof32*,* ColDof32-CalDof 33*,* ColDof32-CalDof39*,* ColDof32-CalDof19*,* ColDof32-CalDof7*,* ColDof33-CalDof41*,* ColDof33-CalDof8*,* ColDof34-CalDof50*,* ColDof34-Ca lDof39*,* ColDof34-CalDof33*, and *ColDof34-CalDof19*. The next most common was that there were 13 homologous gene pairs on Ol4 and Ol10, On Ol4, they were *ColDof8-CalDof25*,* ColDof98-CalDof28*,* ColDof9-CalDof22*,* ColDof9-CalDof26*,* ColDof10-CalDof21*,* ColDof10-CalDof24* and *ColDof3 6-CalDof23*,* ColDof42-CalDof26*,* ColDof42-CalDof22*,* ColDof43-CalDof27*,* ColDof43-CalDof24*,* ColDof43-CalDof21*, and *ColDof22-CalDof44*. On Ol10, they were *ColDof1-CalDof51*,* ColDof2-CalDof53*,* ColDof2-CalDof34*,* ColDof20-CalDof52*,* ColDof20-CalDof31*,* ColDof22-CalDof45 and ColDof 22-CalDof49*,* ColDof22-CalDof18*,* ColDof22-CalDof11*,* ColDof25-CalDof50*,* ColDof25-CalDof39*,* ColDof25-CalDof19*, and *ColDof25-CalDof7*. Least on Ol5, Ol6, Ol8 and Ol14. There were two homologous gene pairs: *ColDof45-CalDof40* and *ColDof45-CalDof29*, *ColDof37-CalDof14* and *ColDof38-CalDof14*,* ColDof15-CalDof41*,* ColDof15-CalDof8* and *ColDof29-CalDof16* and *ColDof13-CalDof29* with no homologous pairs in Ol1, Ol2, and Ol11 (connected by orange lines in Supplementary Fig. 4B).

In order to study the evolutionary history of Dof genes among different teas, the Dof gene family of Yunkang No. 10 of Pu-erh tea was analyzed by collinear analysis. The results showed that 43 pairs of *Dof* gene family members were related to camellia oil. It *had* the most homologous pairs on Ol3, 11 pairs, They were *ColDof3-CaSDof6*,* ColDof5-CaSDof13*,* ColDof31-CaSDof2*,* ColDof31-CaSDof28*,* ColDof32-CaSDof2*,* ColDof32-CaSDof28*,* ColDof6-CaSDof 3*,* ColDof33-CaSDof27*,* ColDof34-CaSDof2*,* and ColDof34-CaSDof28*. Next, there are at most six homologous gene pairs on Ol4 and Ol7, On Chr4, they were *ColDof8-CaSDof31*,* ColDof9¬-CaSDof32*,* ColDof10-CaSDof30*,* ColDof10-CaSDof33*,* ColDof43-CaSDof33*, and *ColDof43-CaSDof30*. On Chr7, they were *ColDof39-CaSDof8*,* Coldof11-Casdof36*,* ColDof11¬-CaSDof7*,* ColDof14-CaSDof37*,* ColDof39-CaSDof33*, and *ColDof39-CaSDof22*. At least one homologous gene pair *ColDof29-CaSDof29* and *ColDof39-CaSDof22* was found on Ol14 and Ol8, and no homologous gene pair was found in Ol1, Ol2, Ol11 and Ol13 (connected by yellow lines in Supplementary Fig. 4C).

The *Dof* gene family of Longjing 43 (*C. sinensis var. sinensis*) was analyzed collinearly. The results showed that 65 pairs of *Dof* gene family members were related to oil tea. It had the most homologous gene pairs on Ol3, with 14 pairs, They were *ColDof3-GWHDof2*,* ColDof3-GWHDof31*,* ColDof5-GWHDof18*,* ColDof31-GWHDof6*,* ColDof32-GWHDof20*,* ColDof32-GWHDof22*,* and ColDof32-GWHDof4*,* ColDof32-GWHDof6*,* ColDof33-GWHDof21*,* ColDof33-GWHDof5*,* ColDof34-GWHDof22*,* ColDof34-GWHDof20*,* ColDof34-GWHDof4* and *ColDof34-GWHDof6*. The next most common was on Ol7, where there were eight homologous gene pairs. They are *ColDof11-GWHDof11*,* ColDof14-GWHDof16*,* ColDof14-GWHDof15*,* ColDof39-GWHDof3*,* ColDof39-GWHDof17*,* ColDof39-GWHDof24*, and *ColDof39-G WHDof8* and *ColDof39-GWHDof13*. At least, there is only one homologous gene pair *ColDof15-GWHDof5* in Ol8, and no homologous gene pair in Ol1, Ol2, Ol11 and Ol14 (linked by red lines in Supplementary Fig. 4D).

Shuchazao was also one of the varieties of tea, and the *Dof* gene family of Shuchazao is collinear analysis. According to the results, 73 pairs of Dof gene family members are related to Camellia oil. It had the most homologous pairs on Ol10, 13, They were *ColDof1-CSSDof37*,* ColDof2-CSSDof42*,* ColDof2-CSSDof3*,* ColDof20-CSSDof26*,* ColDof20-CSSDof36*,* ColDof20-CSSDof19*, and *ColDof22-CSSDof43*,* ColDof22-CSSDof15*,* ColDof22-CSSDof7*,* ColDof22-CSSDof22*,* ColDof22-CSSDof39*,* ColDof25-CSSDof10* and *ColDof25-CSSDof32*. The next most common is on Ol4, where there are 12 homologous gene pairs, They were *ColDof8-CSSDof11*,* ColDof10-CSSDof27*,* ColDof10-CSSDof21*,* ColDof4-CSSDof8*,* ColDof36-CSSDof14*,* ColDof9-CSSDof18*,* and ColDof36-CSSDof25*,* ColDof42-CSSDof14*,* ColDof42-CSSDof18*,* ColDof43-CSSDof27*,* ColDof43-CSSDof21*, and *ColDof44-CSSDof18*. Ol5, Ol6, Ol13 and Ol14 had at least 2 homologous gene pairs. They were *ColDof45-CSSDof17* and *ColDof45-CSSDof30*,* ColDof37-CSSDof35* and *ColDof38-CSSDof35*,* ColDof28-CSSDof10* and *ColDof28-CSSDof32* and *ColDof29-CSSDof36* and *ColDof29-CSSDof40*, with no homologous pairs in Ol1, Ol2, Ol8, and Ol11 (linked by red lines in Supplementary Fig. 4E).

The collinearity analysis of *Dof* gene family of Tieguanyin showed that 85 pairs of *Dof* gene family members were related to *C.oleifera*. It had the most homologous gene pairs on Ol3, with 17 pairs, They were *ColDof3-SIVDof15*,* ColDof3-SIVDof29*,* ColDof5-SIVDof10*,* ColDof5-SIVDof28*,* ColDof31-SIVDof12*,* ColDof31-SIVDof32*,* ColDof32-SIVD of1*,* ColDof32-SIVDof12*,* ColDof32-SIVDof14*,* ColDof32-SIVDof30*,* ColDof32-SIVDof32*,* ColDof33-SIVDof13*,* ColDof33-SIVDof31*,* ColDof34 -SIVDof1*,* ColDof34-SIVDof14*,* ColDof34-SIVDof12*,* ColDof34-SIVDof30 and ColDof34-SIVDof32*. The next most common was on Ol10, where there were 13 homologous gene pairs, They were *ColDof1-SIVDof15*,* ColDof1-SIVDof37*,* ColDof2-SIVDof15*,* ColDof2-SIVDof36*,* ColDof20-SIVDof2*,* ColDof20-SIVDof19*,* and ColDof20-SIVDof34*,* ColDof22-SIVDof7*,* ColDof22-SIVDof16*,* ColDof25-SIVDof1*,* ColDof25-SIVDof12*,* ColDof25-SIVDof30 and ColDof25-SIVDof32*. Ol5, Ol6, Ol8 and Ol14 had at least 2 homologous gene pairs. They were *ColDof45-SIVDof10* and *ColDof45-SIVDof28*,* ColDof37-SIVDof35* and *ColDof38-SIVDof35*,* ColDof15-SIVDof13* and *ColDof15-SIVDof31* and *ColDof29- SIVDof34* and *ColDof29-SIVDof38*, with no homologous pairs in Ol1, Ol2, and Ol11 (linked by red lines in Supplementary Fig. 4F).

The collinearity analysis of *Dof* gene family of Biyun showed that 50 pairs of *Dof* gene family members were related to *C.oleifera*. It had the most homologous gene pairs on Ol3, with 10 pairs, They were *ColDof5-MJRDof10*,* ColDof5-MJRDof6*,* ColDof32-MJRDof3*,* ColDof32-MJRDof7*,* ColDof32-MJRDof17*,* ColDof33-MJRDof20*, and *ColdoF33-MJRRDO f6*,* ColDof34-MJRDof3*,* ColDof34-MJRDof17*, and *ColDof34-MJRDof7*. The next most common was on Ol10, where there were eight homologous gene pairs, They were *ColDof20-MJRDof2*,* ColDof22-MJRDof1*,* ColDof22-MJRDof12*,* ColDof22-MJRDof14*,* ColDof22-MJRDof13*,* ColDof22-MJRDof19*, and *ColDof25-MJ RDof3* and *ColDof25-MJRDof17*. At least one homologous gene pair was found in Ol5 and Ol13, namely *ColDof45-MJRDof10 and ColDof28-MJRDof7*, while no homologous gene pair was found in Ol1, Ol2, Ol11 and Ol14 (linked by red lines in Supplementary Fig. 4G).

In order to show the relationship between *C.oleifera* and tea tree more directly, the *Dof* gene family of tea tree diploid genome was collinear analysis, and 82 pairs of *Dof* gene family members were found to have source relationship with *C.oleifera*. It has the most homologous gene pairs on Ol3, with 16 pairs, They are *ColDof3-DipDof10*,* ColDof3-DipDof29*,* ColDof5-DipDof11*,* ColDof5-DipDof28*,* ColDof32-DipDof1*,* Coldof32-DipDO13*,* and ColDof32-DipDof15*,* ColDof32-DipDof30*,* ColDof32-DipDof32*,* ColDof33-DipDof14*,* ColDof33-DipDof31*,* ColDof34-DipDof1*,* ColDof34-DipDof15*,* ColDof34-DipDof13*,* ColDof34-DipDof30*, and *ColDof34-DipDof32*. The next most common was on Ol12, where there were 12 homologous gene pairs, They were *ColDof8-DipDof27*,* ColDof8-DipDof23*,* ColDof9-DipDof21*,* ColDof9-DipDof25*,* ColDof10-DipDof24*,* ColDof10-DipDof20*, and *ColDof36-DipDof26*,* ColDof42-DipDof21*,* ColDof42-DipDof25*,* ColDof43-DipDof20*,* ColDof43-DipDof24*, and *ColDof44-DipDof25*. At least, there is only one homologous gene pair *ColDof29-DipDof34* on Ol14, and no homologous gene pair in Ol1, Ol2 and Ol11 (linked by red lines in Supplementary Fig. 4H).

By collinear analysis of *Dof* gene family in tea haploid genome, 148 pairs of *Dof* gene family members were derived from *C.oleifera*. It had the most homologous gene pairs on Ol3, with 29 pairs, They were *ColDof3-HapDof16*,* ColDof3-HapDof54*,* ColDof3-HapDof22*,* ColDof5-HapDof17*,* ColDof5-HapDof53*,* ColDof5-HapDof23*,* and ColDof5-HapDof55*,* ColDof31-HapDof19*,* ColDof31-HapDof60*,* ColDof32-HapDof19*,* ColDof32-HapDof21*,* ColDof32-HapDof56*,* ColDof32-HapDof58*,* ColDof32-HapDof3*,* ColDof32-HapDof25*,* ColDof32-HapDof27*,* ColDof32-HapDof57*,* ColDof32-HapDof60*,* ColDof33-HapDof20*,* ColDof33-HapDof26*,* ColDof33-HapDof59*,* ColDof34-HapDof21*,* ColDof34-HapDof19*,* ColDof34-HapDof56*,* ColDof34-HapDof58*,* ColDof34-HapDof27*,* ColDof34-HapDof25 ColDof34-HapDof57* and *ColDof34-HapDof60*. The next most common was on Ol10, where there were 25 homologous gene pairs, They were *ColDof1-HapDof16*,* ColDof1-HapDof66*,* ColDof1-HapDof22*,* ColDof1-HapDof67*,* ColDof2-HapDof2*,* ColDof2-HapDof16*,* ColDof2-HapDof6*,* ColDof20-HapDof1*,* ColDof20-HapDof35*,* ColDof20-HapDof62*,* ColDof20-HapDof4*,* ColDof20-HapDof33*,* ColDof20-HapDof36*,* ColDof20-HapDof64*,* ColDof22-HapDof8*,* ColDof22-HapDof29*,* ColDof22-HapDof61*,* ColDof22-HapDof32*,* Coldof25-Hapdof19*,* Coldof25-hapdof56*,* ColDof25-HapDof58*,* ColDof25-HapDof3*,* ColDof25-HapDof25*,* ColDof25-HapDof57*, and *ColDof25-HapDof60*. At least on Ol6, there were 2 homologous pairs *ColDof37-HapDof65* and *ColDof38-HapDof65*, and no homologous pairs in Ol1, Ol2, and Ol11 (linked by red lines in Supplementary Fig. 4I).

**Fig. 7:**
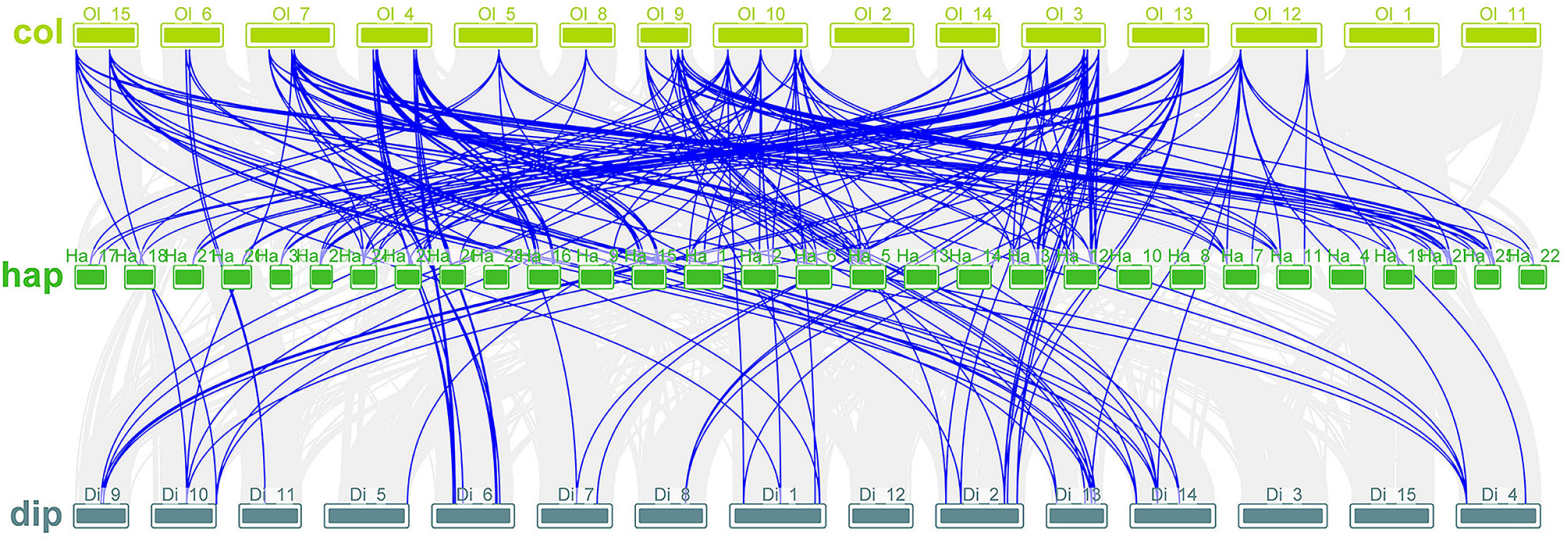
Collinearity analysis of *Dof* gene family between *Camellia oleifa*(Ol_1-Ol_15) and *Camellia sinensis* HD haplotype genome (Ha_1-Ha_30)、*Camellia sinensis* HD diploid genome (Di_1-Di_15)

In order to further infer the origin and evolutionary mechanism of *ColDof* gene in *C. oleifolia*, the homology relationship between 45 *ColDofs* and other Camellia species was studied. These species include the haploid genome, diploid genome, Xiangye Camellia, Yunkang 10, Longjing 43, Shuchazao, Tieguanyin and Biyun, which have obvious collinearity with *C. oleifera*. It is worth noting that *ColDofs* of *C. oleifolia*, tea tree haploid genome and tea tree diploid genome were significantly stronger than other species, which may be related to the closely related species of *C. oleifolia*, tea tree haploid genome and tea tree diploid genome (Fig. 7).

### Molecular evolution analysis of ColDof proteins

**Fig. 8:**
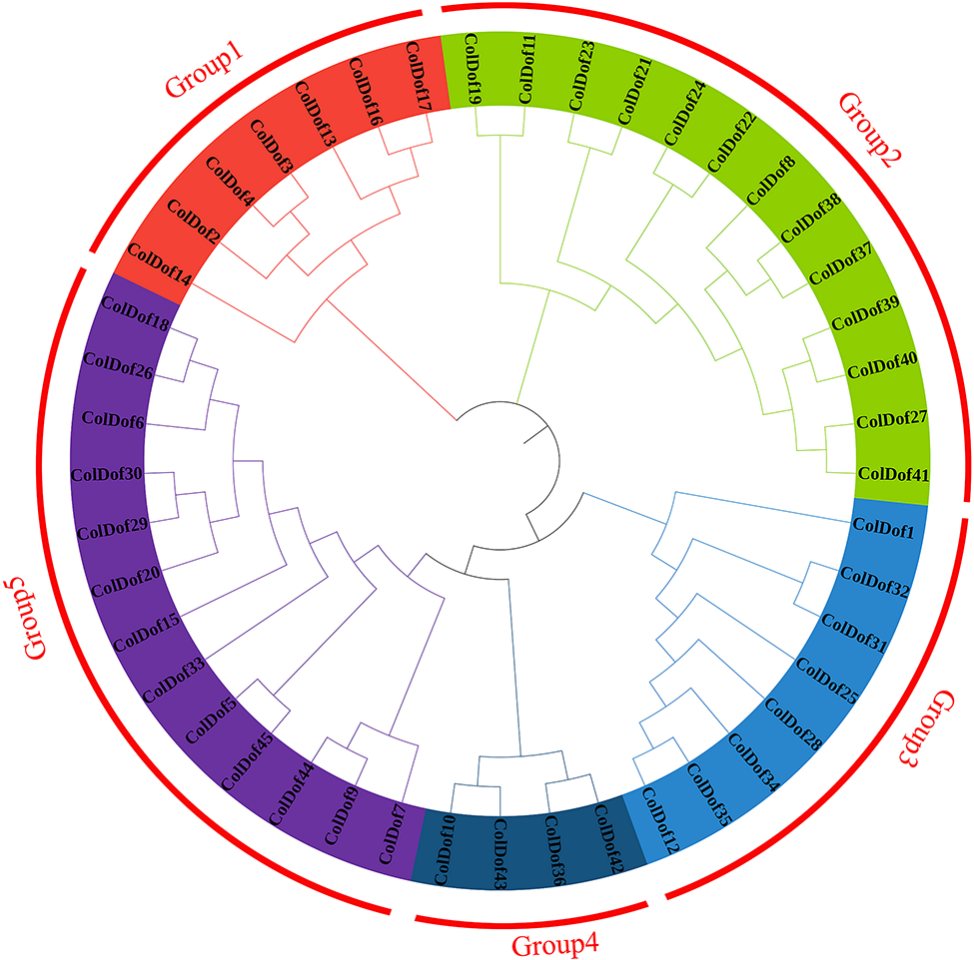
Phylogenetic tree of Dof proteins in *C.oleifera*

Through the evolutionary tree composed of 45 ColDof proteins, we can see that 45 ColDof proteins were clearly divided into 5 groups according to their degree of aggregation in the evolutionary tree (thus labeled Group1, Group2, Group3, Group4, Group5). In the whole family, Group1 had the most primitive evolutionary speed, including 7 ColDof proteins, among which ColDof14 had the slowest evolution, ColDof16 and ColDof17 had the fastest evolution. The second group with the fastest evolution speed was Group2, which contains 13 ColDof proteins, among which ColDof19 and ColDof11 evolve the slowest, and ColDof41 and ColDof27 evolve the fastest. Group5 was the fastest evolving branch, comprising 13 ColDof proteins, of which ColDof17 was the slowest, and ColDof18 and ColDof26 were the fastest (Fig. 8).

The phylogenetic tree of ColDof and AtDof gene families of *Arabidopsis thaliana* was divided into 7 subbranches (thus labeled Group1, Group2, Group3, Group4, Group5, Group6, Group7), each containing 2–46 members. In the whole family, Group1 was the most primitive evolutionary branch, including 4 members, including 1 ColDof member and 3 AtDof members, and ColDof35 was the slowest evolution; Second, Group2 had 7 members, including 5 ColDof members and 2 AtDof members. ColDof28 was the slowest, ColDof25 was the fastest, and ColDof34 was the same. Group3 contained two ColDof members and two AtDof members, and ColDof36 was the slowest evolution, AtDof6 and AtDof7 were the fastest evolution; Group4 contained 12 ColDof members and 15 AtDof members, and ColDof15 was the slowest in evolution, AtDof42, ColDof18, ColDof26 and ColDof6 with similar evolutionary speed, and ColDof30 was the fastest in evolution. At the same rate of evolution is AtDof37; Group5 contains two AtDof members, AtDof14 and AtDof15; Group6 contained ColDof1 and AtDof46; Finally, Group7 was the fastest evolving branch, including 24 ColDof members and 22 AtDof members, and ColDof12 was the slowest evolving, ColDof27 was the fastest evolving, and AtDof40 and AtDof41 were similar to its evolving speed. Overall, Group1 was the most original, while Group6 was the fastest growing (Supplementary Fig. 5A).

In order to further study the evolutionary relationship of *Dof* genes in *C.oleifera*, the phylogenetic trees of *ClaDof* and *ColDof* gene families were divided into 6 subbranches (thus labeled Group1, Group2, Group3, Group4, Group5, Group6). Each subbranch contains 6–41 members. In the whole family, Group1 had the most primitive evolutionary speed, including 12 members, including 4 ColDof members and 8 ClaDof members, and ColDof15 had the slowest evolutionary speed, and ClaDof8 and ColDof15 had the same slow evolutionary speed. Group2 had 25 members, including 10 ColDof members and 15 ClaDof members, and ColDof20 had the slowest evolution; ClaDof52 and ColDof52 had the same slow evolution speed; ColDof45 had the fastest evolution speed. ClaDof20, ClaDof40, ClaDof35 and ClaDof38 had similar evolutionary speed. Group3 contained 3 ColDof members and 4 ClaDof members, and ColDof18 was the slowest in evolution, ClaDof9 and ColDof18 had the same slow evolution speed, ColDof26 had the fastest evolution speed, and ClaDof2 and ClaDof47 had similar evolution speed. Group4 contained 3 ColDof members and 3 ClaDof members, and ColDof7 was the slowest in evolution, ClaDof26 was the same as its evolution speed, and the fastest in evolution were ColDof9, ColDof44, ClaDof6 and ClaDof22. Group5 contained 4 ColDof members and 5 ClaDof members, and ColDof43 was the slowest in evolution, ClaDof24 was the same as ColDof43 in evolution speed, and ColDof36 was the fastest in evolution speed, and ClaDof5 and ClaDof23 were similar in evolution speed. Finally, Group6 had the fastest evolutionary speed, including 21 ColDof members and 20 ClaDof members, and ColDof1 was the slowest evolutionary speed, ClaDof51 and ColDof1 had the same evolutionary speed, ColDof8 had the fastest evolutionary speed, ClaDof22 had the same evolutionary speed. Overall, Group1 was the most original, while Group6 was the fastest growing (Supplementary Fig. 5B).

To study the evolutionary relationship of *Dof* genes among different teas, The phylogenetic tree of *ColDof* and *CasDof* gene families of Pu-erh tea was divided into 8 subbranches (thus labeled Group1, Group2, Group3, Group4, Group5, Group6, Group7, Group8), each containing 1–36 members. In the whole family, Group1 was the most primitive evolutionary branch, including one member ColDof1; Group2, which has two ColDof members and one CaSDof member, had the slowest evolution, and CaSDof16 had the slowest evolution, while ColDof45 and ColDof36 had the fastest evolution. Group3 contains 5 ColDof members and 5 CaSDof members, and ColDof5 was the slowest in evolution, CaSDof4 was the same as ColDof5 in evolution speed, and ColDof29 was the fastest in evolution speed, and CaSDof29 was the same in evolution speed. Group4 contained two ColDof members and three CaSDof members, and ColDof15 was the slowest in evolution, CaSDof39 was the same as the slow in evolution, ColDof33 was the fastest in evolution, CaSDof27 and CaSDof1 were the same in evolution. Group5 contained 3 ColDof members and 1 CaSDof member, and ColDof44 and ColDof9 were the slowest in evolution, ColDof7 was the fastest in evolution, and CaSDof32 was the same in evolution speed. Group6 contained 10 ColDof members and 9 CaSDof members, and ColDof26 was the slowest in evolution, CaSDof19 was the same as ColDof26 in evolution speed, and ColDof31 and ColDof32 were the fastest in evolution. Just as fast are CaSDof2 and CaSDof26; Group7 contained two ColDof members and three CaSDof members, and ColDof10 has the slowest evolution, CaSDof30 has the same slow evolution, ColDof43 had the fastest evolution, and CaSDof33 has the same fast evolution. Finally, Group8 was the fastest evolving branch, which contained 20 ColDof members and 17 CaSDof members, and ColDof11 was the slowest evolving branch, CaSDof36 and ColDof11 are the same evolving speed. The most rapidly evolving were ColDof21, ColDof22, ColDof23 and ColDof24, with CaSDof38 evolving just as fast. In summary, ColDof1 was the most original team, while Group8 was the fastest growing team (Supplementary Fig. 5C).

The phylogenetic tree of *ColDof* and *GWHDof* gene families of *C. sinensis var. Longjing 43* was divided into 8 subbranches (thus labeled Group1, Group2, Group3, Group4, Group5, Group6, Group7, Group8). Each subbranch contained 2–37 members. In the whole family, Group1 was the most primitive evolutionary speed, including two ColDof members, ColDof31 and ColDof32, and one GWHDof member, GWHDof20, and all three members evolve at the same slow speed. Group2, which had two ColDof members and two GWHDof members, had the slowest evolution, and GWHDof6 had the slowest evolution, while ColDof25 and ColDof34 had the fastest evolution, and GWHDof22 had the same evolution speed. Group3 contained a ColDof member, ColDof28, and a GWHDof member, GWHDof4, both of which evolve at the same speed. Group4 contains one ColDof member and two GWHDof members, and GWHDof1 was the slowest in evolution, ColDof1 was the fastest in evolution, and GWHDof2 was the same in evolution speed. Group5 contained two ColDof members, and ColDof35 and ColDof12 evolve at the same speed. Group6 contained 13 ColDof members and 12 GWHDof members, and ColDof29 and ColDof30 were the slowest in evolution, and GWHDof30, GWHDof7 and ColDof20 were similar in evolution speed, and ColDof7 was the fastest in evolution. Just as fast were GWHDof33 and GWHDof34; Group7 contains two ColDof members and two GWHDof members, and ColDof42 was the slowest in evolution, GWHDof29 was the same in evolution, ColDof36 was the fastest in evolution, and GWHDof26 is the same in evolution speed. Finally, Group8 was the fastest evolving group, consisting of 22 ColDof members and 15 GWHDof members, and ColDof11 was the slowest evolving group, with similar evolutionary speed including GWHDof12, GWHDof11 and ColDof19. The most rapidly evolving were ColDof37 and ColDof38, and as rapidly evolving are GWHDof9; In summary, ColDof1 was the most original team, while Group8 was the fastest growing team (Supplementary Fig. 5D).

The phylogenetic tree of *ColDof* and *CSSDof* gene families of *Camellia sinensis var. sinensis* cv. Shuchazao was divided into 6 subbranches (labeled Group1, Group2, Group3, Group4, Group5, Group6), each containing 1–40 members. In the whole family, Group1 had the most primitive evolutionary speed, including 10 members, including 7 ColDof members and 3 CSSDof members, and ColDof28 was the slowest evolutionary speed, and CSSDof32 was the same as its evolutionary speed. Group2, which has 31 members, including 15 ColDof members and 16 CSSDof members, and ColDof20, which had the slowest evolution, had similar evolutionary speed with CSSDof36, CSSDof40, ColDof30 and ColDof29. ColDof5 evolved the fastest, while CSSDof17 evolved at the same rate. Group3 contained one CSSDof member, CSSDof23. Group4 contained one ColDof member, ColDof1, and one CSSDof member, CSSDof37, both of which evolve at the same speed. Group5 contained two ColDof members and two CSSDof members, and ColDof19 was the slowest in evolution, CSSDof12 and ColDof19 have the same slow evolution speed, and ColDof11 had the fastest evolution speed, and CSSDof41 has the same evolution speed. Finally, Group6 was the fastest evolutionary group, which contained 20 ColDof members and 20 CSSDof members, and ColDof13 was the slowest evolutionary group, and its evolutionary speed was similar to that of CSSDof1, ColDof17 and ColDof16. The most rapidly evolving were ColDof37 and ColDof38, with CSSDof35 and CSSDof38 evolving just as fast (Supplementary Fig. 5E).

The phylogenetic tree of *ColDof* and *SIVDof* gene families of *Camellia sinensis var. Sinensis* Tieguanyin was divided into 6 subbranches (thus labeled Group1, Group2, Group3, Group4, Group5, Group6), each containing 3–37 members. In the whole family, Group1 had the most primitive evolutionary speed, including 4 members, including 2 ColDof members and 2 SIVDof members, and ColDof33 had the slowest evolution, and SIVDof13 and ColDof33 had the same evolutionary speed. Group2 has 19 members, including 8 ColDof members and 11 SIVDof members, and ColDof18 had the slowest evolution, SIVDof41 had the same slow evolution as ColDof18, and ColDof29 has the fastest evolution. SIVDof39 and SIVDof38 had similar evolutionary speed. Group3 contained 8 ColDof members and 8 SIVDof members, and ColDof1 was the slowest in evolution, SIVDof37 was the same as ColDof1 in evolution speed, and ColDof25 was the fastest in evolution speed, and SIVDof1 is the same in evolution speed. Group4 contains two ColDof members and one SIVDof member, and ColDof36 was the slowest in evolution, while ColDof42 and SIVDof22 are the fastest in evolution. Group5 contains 5 ColDof members and 4 SIVDof members, and ColDof11 was the slowest in evolution, SIVDof6 and ColDof11 had the same slow evolution speed, ColDof9 had the fastest evolution speed, and SIVDof9 had the same evolution speed. Finally, Group6 was the fastest evolving group, containing 20 ColDof members and 17 SIVDof members, and ColDof13 was the slowest evolving group, with similar evolutionary speed including SIVDof40, ColDof16 and ColDof17. The most rapidly evolving were ColDof21 and ColDof23; Overall, Group1 was the most original, while Group6 was the fastest growing (Supplementary Fig. 5F).

The phylogenetic trees of *ColDof* and *MJRDof* gene families were divided into 10 subbranches (thus labeled Group1, Group2, Group3, Group4, Group5, Group6, Group7, Group8, Group9, Group10). Each subbranch contains 1–29 members. In the whole family, Group1 was the most primitive evolutionary branch and contained one MJRDof member. The next group that evolved faster was Group2, which included one ColDof member, ColDof18. Group3 contained two ColDof members and two MJRDof members, and ColDof6 was the slowest in evolution, MJRDof5 is the same as ColDof6 in evolution speed, ColDof26 was the fastest in evolution speed, and MJRDof4 is the same in evolution speed. Group4 contained three ColDof members and one MJRDof member, and ColDof30 and ColDof29 evolve the slowest, while ColDof20 and MJRDof2 evolve the fastest. Group5 contained one ColDof member and one MJRDof member, and MJRDof20 evolves at the same speed as ColDof15. In Group6, there were 6 ColDof members and 2 MJRDof members, and ColDof7 was the slowest in evolution, ColDof44 and ColDof9 were similar in evolution speed, ColDof45 was the fastest in evolution, and MJRDof15 was the same in evolution speed. There were 8 ColDof members and 6 MJRDof members in Group7, and ColDof36 was the slowest in evolution, MJRDof8 was the same in evolution speed, ColDof25 was the fastest in evolution speed, MJRDof3 was the same in evolution speed. Group8 contained 3 ColDof members and 1 MJRDof member, and ColDof1 was the slowest evolution, while ColDof11 and MJRDof16 were the fastest evolution. Group9 contains 10 ColDof members and 1 MJRDof member, and ColDof43 was the slowest in evolution, MJRDof9 and ColDof10 were similar in evolution speed, and ColDof16 and ColDof17 were the fastest in evolution speed. Finally, Group10 was the fastest evolving group, including 11 ColDof members and 8 MJRDof members, and ColDof40 was the slowest evolving group, with the same evolutionary speed as MJRDof22, and the fastest evolving group was ColDof21. As fast as ColDof21 which was evolving, was MJRDof13. Overall, Group1 was the most original, while Group10 is the fastest growing (Supplementary Fig. 5G).

In order to more intuitively show the evolutionary relationship between oil tea and tea tree, The phylogenetic tree of the tea diploid genome *ColDof* and *DipDof* gene families was divided into 8 subbranches (thus labeled Group1, Group2, Group3, Group4, Group5, Group6, Group7, Group8), each containing 1–41 members. In the whole family, Group1 was the most primitive evolutionary speed, including one DipDof member DipDof8; Second, Group2 had a slightly faster evolutionary speed, including two ColDof members and one DipDof member. Among them, ColDof11 had the fastest evolutionary speed, and ColDof19 had the slowest evolutionary speed, and DipDof41 had the same evolutionary speed. Group3 contained one ColDof member, ColDof1, and one DipDof member, DipDof36. Group4 contained 5 ColDof members and 2 DipDof members, and ColDof14 and DipDof9 evolve the slowest, while ColDof16 and DipDof38 evolve the fastest. Group5 contained two ColDof members and two DipDof members, and DipDof10 evolves as fast as ColDof3. Group6 contains 3 ColDof members and 3 DipDof members, and ColDof2 was the slowest evolving, DipDof4 is the same evolving speed, ColDof10 was the fastest evolving speed, DipDof24 was the same evolving speed. There are 11 ColDof members and 9 DipDof members in Group7, and ColDof40 was the slowest in evolution, and DipDof40 was the same in evolution speed, ColDof27 was the fastest in evolution speed, and DipDof35 is the same in evolution speed. Group8 contained two ColDof members and two DipDof members, and ColDof42 and DipDof22 evolve the slowest, while ColDof36 and DipDof26 evolve the fastest. In Group9, there were 6 ColDof members and 5 DipDof members, and ColDof7 was the slowest in evolution, DipDof21 was the same in evolution speed, and ColDof5 and DipDof11 were the fastest in evolution. Finally, Group10 was the fastest evolving group, including 13 ColDof members and 15 DipDof members, and ColDof15 was the slowest evolving group, which had the same evolutionary speed as DipDof31, and the fastest evolving group is ColDof31. As fast as ColDof31, which was evolving was DipDof13. Overall, Group1 was the most original, while Group10 was the fastest growing (Supplementary Fig. 5H).

The phylogenetic trees of *ColDof* and *HapDof* gene families were divided into 8 subbranches (thus labeled Group1, Group2, Group3, Group4, Group5, Group6), each containing 6–54 members. In the whole family, Group1 has the most primitive evolutionary speed, including 13 members, including 3 ColDof members and 10 HapDof members, and ColDof20 had the slowest evolution, ColDof30 had the fastest evolution speed, and HapDof64 and HapDof62 had the same evolutionary speed. Group2 had 16 members, including 6 ColDof members and 10 HapDof members. ColDof28 is the slowest, and HapDof56 and HapDof57 had the same speed of evolution. The most rapidly evolving were ColDof31, ColDof32, HapDof19, and HapDof25. In Group3, there were 5 ColDof members and 10 HapDof members, and ColDof36 was the slowest in evolution, and HapDof43 and HapDof51 were similar in evolution speed, and ColDof6 was the fastest in evolution. HapDof18 and HapDof24 evolve at a similar rate. In Group4, there were 7 ColDof members and 11 HapDof members, and ColDof15 was the slowest, with the same evolutionary speed as HapDof59, and ColDof5 was the fastest, with the same evolutionary speed as HapDof17 and HapDof23. Group5 contained two ColDof members and four HapDof members, and ColDof11 was the slowest to evolve, with HapDof73, HapDof77 and ColDof19 evolving equally fast. Finally, Group6 is the fastest evolving team, including 22 ColDof members and 32 HapDof members, and ColDof1 was the slowest evolving team, with the same evolutionary speed as HapDof66 and HapDof67. The most rapidly evolving were ColDof41, HapDof61, and HapDof63; Overall, Group1 was the most original, while Group6 was the fastest growing (Supplementary Fig. 5I).

**Fig. 9:**
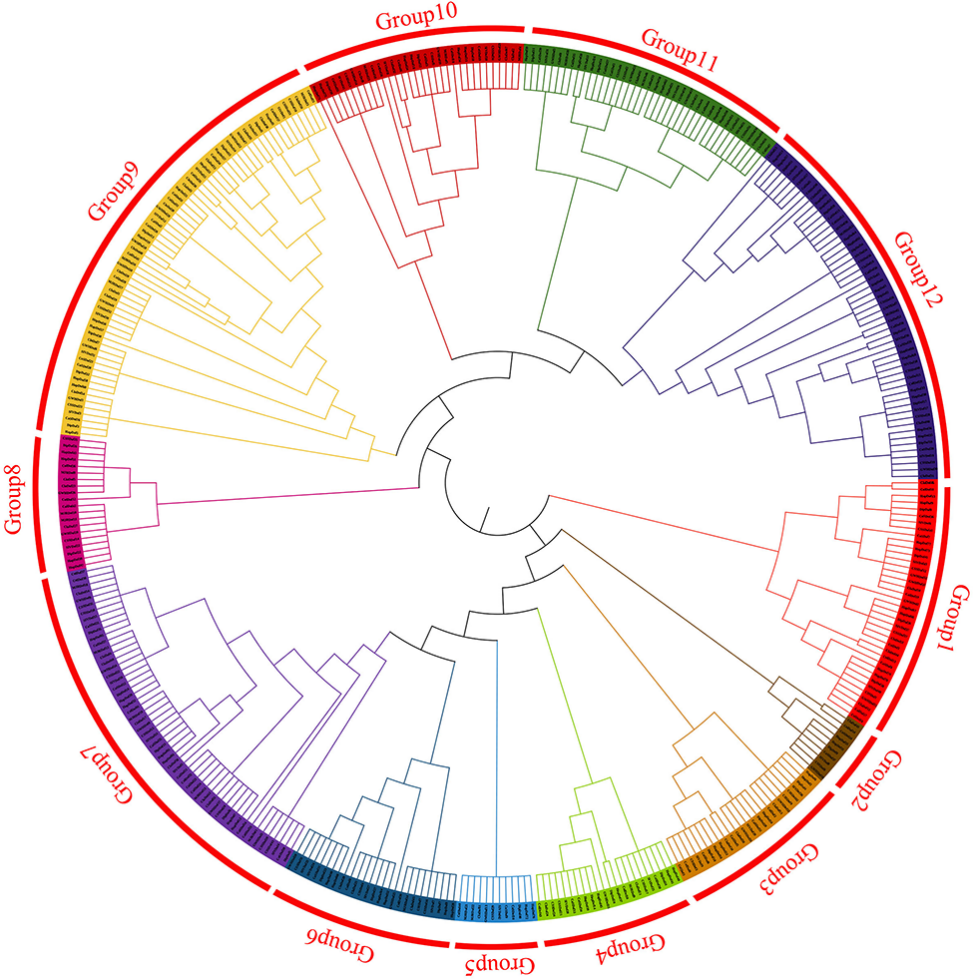
Phylogenetic tree of Dof gene family in *Camellia oleifa* and other multi-species

Based on phylogenetic trees of Dof proteins from *C.oleifera* and other species, The phylogenetic relationships of Dof proteins between cameltea and other species can be divided into 12 groups (thus labeled Group1, Group2, Group3, Group4, Group5, Group6, Group7, Group8, Group9, Group10, Group11, Group12). As can be seen from the Fig., Group1 was the slowest evolving group, which contained 6 members of ColDof. The slowest evolving group is ColDof11, the same evolving speed is ClaDof46, and the fastest evolving group was ColDof16 and ColDof17. HapDof47, HapDof40, DipDof38, SIVDof40, CSSDof1, GWHDof15, and ClaDof12 are all evolving equally fast. There was one ColDof member in Group2. ColDof14 had the slowest evolutionary speed, and ClaDof43 had the same evolutionary speed. There were two ColDof members in Group3. ColDof2 had the slowest evolutionary speed, and HapDof6, HapDof2, DipDof4, SIVDof36, CSSDof9, CSSDof42, GWHDof32 and ClaDof53 had the same evolutionary speed. ColDof3 and ColDof2 evolve the fastest, and HapDof16, HapDof22, DipDof10, SIVDof9, CSSDof3 and CaSDof6 evolve at the same speed. There were two ColDof members in Group4, among which ColDof10 was the slowest to evolve, and HapDof49, HapDof41, DipDof24, SIVDof25, CaSDof30, CSSDof27, MJRDof9 and ClaDof21 were the same to evolve slowly. ColDof43 is the fastest evolving, and HapDof45, HapDof4, HapDof10, DipDof10, SIVDof20, CaSDof33, CSSDof21, CSSDof24, and CaSDof34 are the same. Group5 contained 12 Dof members, all evolving at the same rate; There were two ColDof members in Group6, among which ColDof39 had the slowest evolution, and HapDof28, HapDof8, DipDof7, SIVDof7, CSSDof7, CSSDof16, MJRDof14 and ClaDof45 had the same evolution speed. ColDof8 was the fastest evolving, HapDof44, HapDof52, DipDof27, SIVDof27, CSSDof4 and ClaDof25 were the same evolving speed. In Group7, ColDof had the largest number of members, with 8. The slowest evolving species were ColDof21, ColDof22, ColDof23, ColDof24, HapDof12, HapDof7, DipDof16, GWHDof17, CSSDof20, MJRDof13, ClaDof55, and ClaDof42. The fastest evolving species were ColDof37, ColDof38, HapDof65, DipDof35, DipDof16, GWHDof9, CaSDof21, SIVDof35, CSSDof38, CSSDof35, MJRDof18 and ClaDof14. There were three ColDof members in Group8, and the slowest ones were ColDof42, HapDof39, HapDof47, DipDof22, SIVDof22, GWHDof29, CSSDof14, MJRDof11, ClaDof27 and MJRDof10. ColDof32 had the fastest evolutionary speed, and ColDof36, HapDof51, HapDof43, DipDof26, GWHDof26, CSSDof25, MJRDof8, ClaDof5 and ClaDof23 have similar evolutionary speed. There were 7 ColDof members in Group9, and the slowest ones were ColDof28, HapDof56, HapDof57, SIVDof30, GWHDof4, CSSDof32, MJRDof17, ClaDof19 and ClaDof1. ColDof25 had the fastest evolutionary speed, and SIVDof1, HapDof3, CaSDof17, GWHDof26, CSSDof10, MJRDof3 and ClaDof50 had similar evolutionary speed. There are three ColDof members in Group10, and the ones with the slowest evolution speed were ColDof18, HapDof75, DipDof39, CaSDof35, SIVDof41, GWHDof14, CSSDof13, MJRDof23 and ClaDof9. ColDof6 had the fastest evolutionary speed and ClaDof32 has the same evolutionary speed. There were 5 ColDof members in Group11. The slowest ones were ColDof15, HapDof59, DipDof31, SIVDof31, GWHDof5, CaSDof39, MJRDof20 and ClaDof8, and the fastest ones were ColDof7. HapDof46, HapDof38, DipDof21, SIVDof21, CaSDof32, GWHDof34, CSSDof31, GWHDof33, and ClaDof26 evolved at the same speed. There were 4 ColDof members in Group12. The slowest ones were ColDof5, HapDof23, HapDof17, DipDof11, SIVDof10, GSSDof17 and CaSDof4, and the fastest ones were ColDof29. HapDof69, HapDof68, SIVDof38, CSSDof40, and ClaDof13 evolved at the same rate (Fig. 9).

### Protein-protein interaction analysis of ColDof protein family

**Fig. 10:**
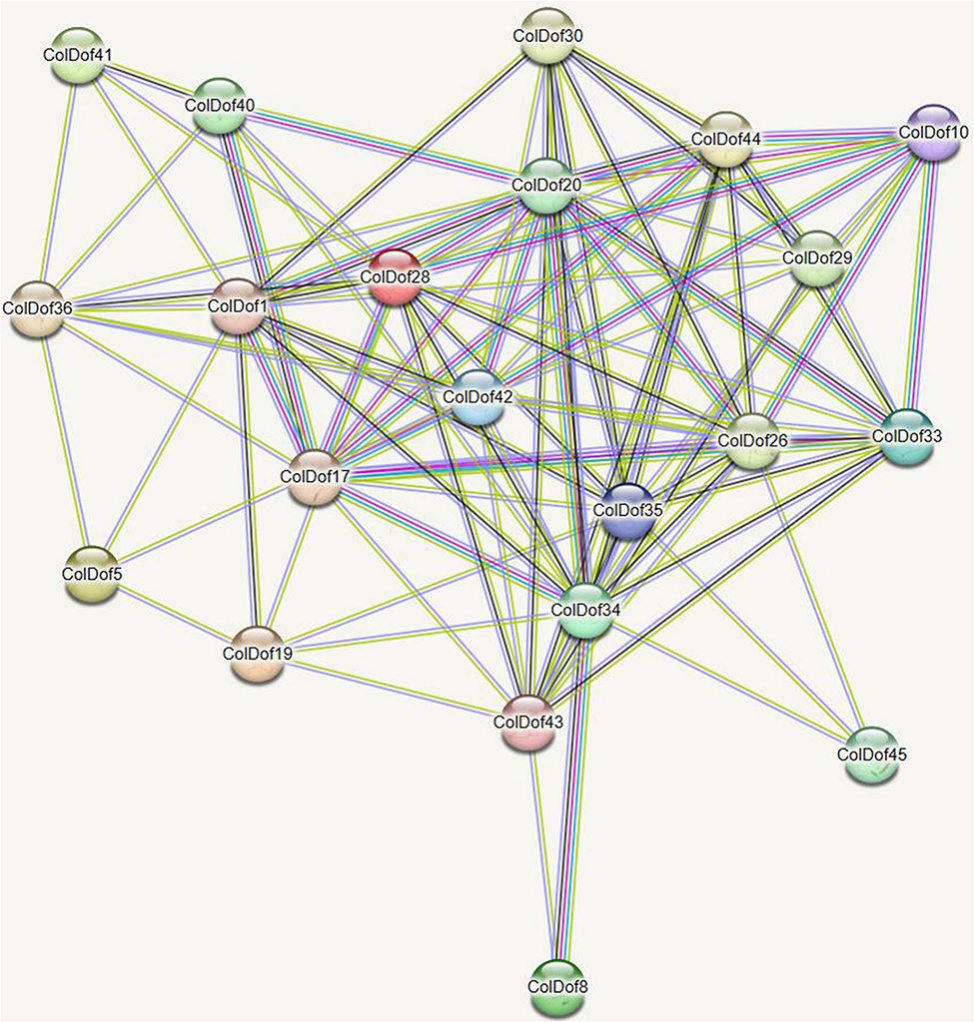
Protein-protein interaction analysis of ColDof proteins

Import ColDof protein sequence file into String (https://string-db.org/) Perform protein-protein interaction prediction in the database, set the species to “*Arabidopsis thaliana*”, save the network file in TSV format, import the TSV file into Cytoscape 3.8.2 software to draw the protein-protein interaction network, perform topology analysis on the network, reflect the size and color of the target with degree values, and reflect the thickness of the edges with combined score values, thereby constructing a protein-protein interaction network. The network consists of 21 nodes and 101 edges, with ColDof34, ColDof20, ColDof28, ColDof35, ColDof42, and ColDof26 as core targets. And ColDof34 had the most protein interactions, with 16 edges, including ColDof8, ColDof35, ColDof17, ColDof45, ColDof43, ColDof20, ColDof29, ColDof44, ColDof30, ColDof19, ColDof40, ColDof26, ColDof42, ColDof28, ColDof33 and ColDof1; Secondly, the protein interaction relationship was most likely ColDof20, with 15 edges, including ColDof35, ColDof17, ColDof43, ColDof29, ColDof28, ColDof1, ColDof36, ColDof10, ColDof44, ColDof40, ColDof33, ColDof26, ColDof42, ColDof30, and ColDof34; ColDof8 had the least interaction with only 2 edges, namely ColDof43 and ColDof34. Protein-protein interaction analysis showed that ColDof34, ColDof20, ColDof28, ColDof35, ColDof42 and ColDof26 have the most protein interactions (Fig. 10).

### Gene expression analysis of *ColDof* genes under 221 *C.oleifera* seed transcriptome and different stress conditions by qRT-PCR experiment

**Fig. 11:**
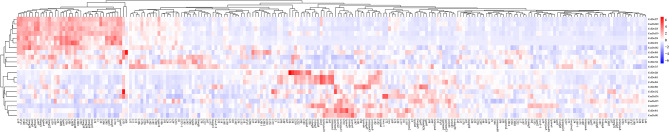
Gene expression analysis of *ColDof* genes under 221 *C.oleifera* seed transcriptome

The transcriptome sequencing analysis of 221 *C. oleifera* varieties showed that 21 *ColDof* genes were expressed in all 221 *C. oleifera* seed species out of 45 *ColDof* gene family members, including *CanDof39*,* ColDof27*,*ColDof25*,* ColDof24*,* CanDof42*,*ColDof21*,*ColDof03*,* ColDof04*,* CanDof40*,* ColDof20*,* ColDof17*,* ColDof19*,* CanDof35*,* ColDof06*,* ColDof16*,* CanDof36*,* CanDof41*,* ColDof32*,* CanDof37*,* ColDof01* and *ColDof09*, while 24 *ColDof* gene members were not expressed. Among the 21 *ColDof* gene members expressed in 221 *C. oleifera* seeds, the *ColDof* gene with the highest expression content was the *ColDof20* gene in L26 *C. oleifera* seeds (expression content of 138.84), while the *ColDof* gene with the lowest expression content was the *ColDof24* gene in Lminyou8 *C. oleifera* seeds (expression content of 0.04)(Fig. 11).

**Fig. 12:**
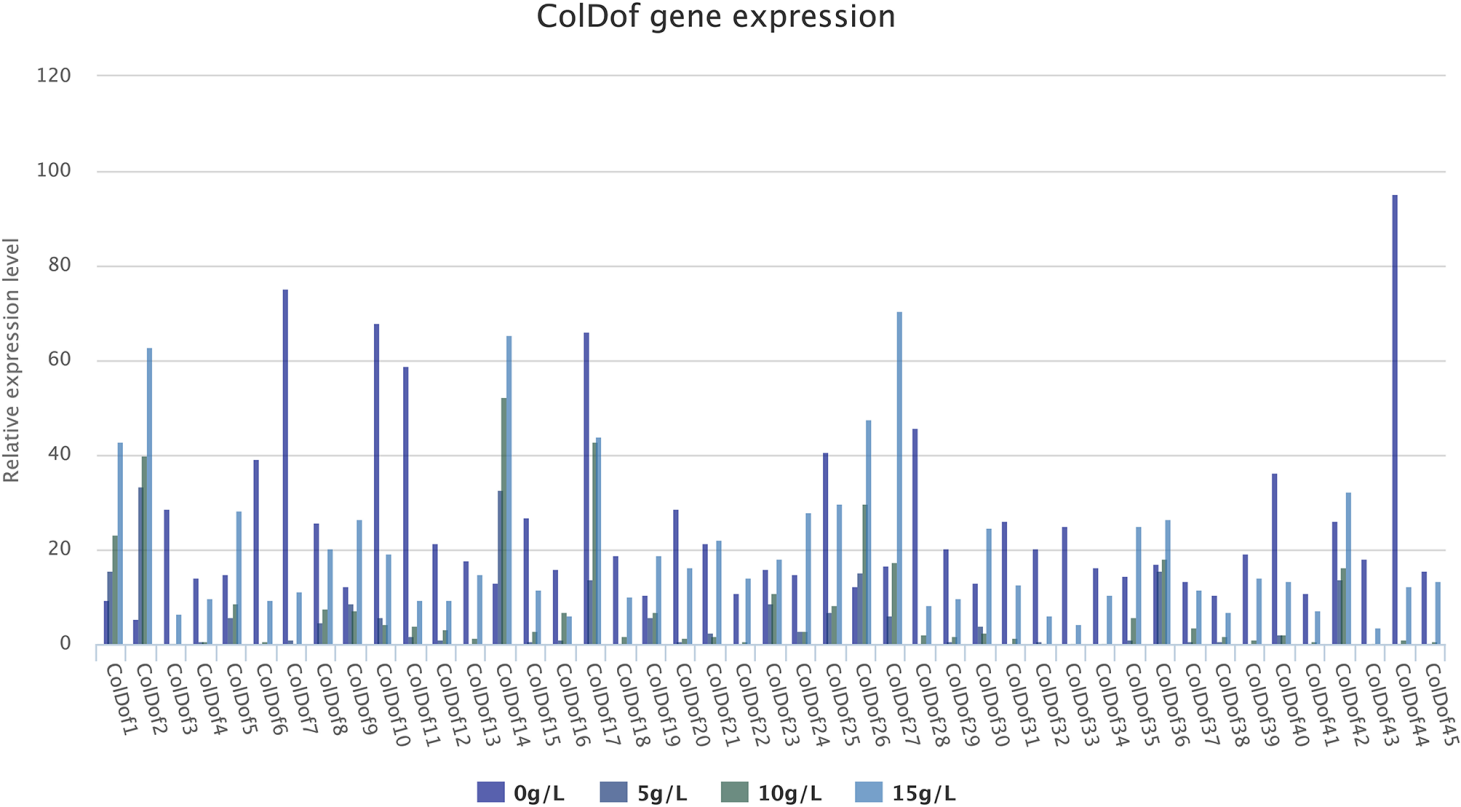
Gene expression analysis of *ColDof* genes under different concentration Nacl by qRT-PCR experiment

The qRT-PCR results of the leaves of *C. oleifera* treated with NaCL solutions of different concentrations(5 g/L, 10 g/L,15 g/L) for 72 h showed that 45 *ColDof* genes were expressed in both the control group and the experimental group. Compared with the control group (without NaCl solution treatment:0 g/L), as the concentration of NaCl solution in the treatment group increased, the expression levels of most *ColDof* genes significantly decreased, while only the expression levels of *ColDof1*,* ColDof2*,* ColDof14* and *ColDof36* genes significantly increased. This indicates that *ColDof1*,* ColDof2*,* ColDof14* and *ColDof36* may be involved in the response to salt stress in *C. oleifera* (Fig. 12).

**Fig. 13:**
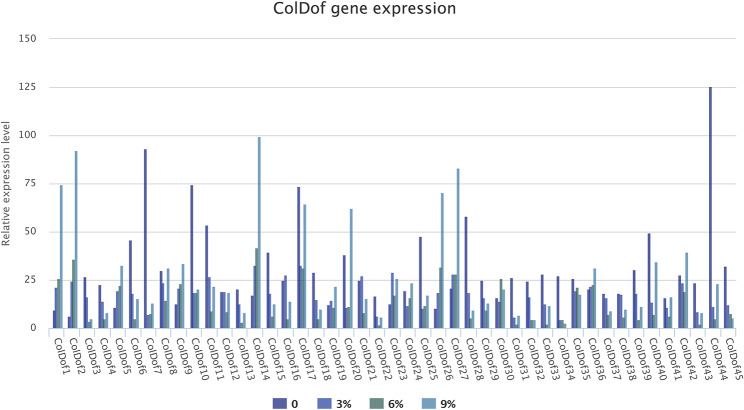
Gene expression analysis of *ColDof* genes under different concentration PEG6000 by qRT-PCR experiment

The qRT-PCR results of the leaves of *C. oleifera* treated with PEG6000 solutions of different concentrations(3%, 6%, 9%) for 72 h showed that 45 *ColDof* genes were expressed in both the control group and the experimental group. Compared with the control group (without PEG6000 solution treatment:0%), as the concentration of PEG6000 solution in the treatment group increased, the expression levels of most *ColDof* genes significantly decreased, while only the expression levels of *ColDof1*,* ColDof2*,* ColDof5 ColDof14*,*ColDof27* and *ColDof36* genes significantly increased. This indicates that *ColDof1*,* ColDof2*,* ColDof5 ColDof14*,*ColDof27* and *ColDof36* may be involved in the response to drought stress in *C. oleifera* (Fig. 13).

## Discussion

The regulation of gene expression is a kind of gene expression regulation for plants to cope with stress and plays a crucial role in plant growth and development, and transcription factors are the most basic regulatory elements. Plant transcription factors have been the focus of functional genomics research. As a class of transcription factors unique to plants, Dof transcription factors interact with cis-elements of specific target genes to regulate various signaling pathways. Compared with 45 ColDofs in oil tea, 24 Dofs were identified in castor seeds [51], 36 Dofs were identified in Arabidopsis [52], and 76 Dofs were identified in Chinese cabbage [53]. The number of Dof family varies greatly in different plants. At present, *Dof* genes are mainly reported in herbaceous plants, and less reported in woody plants. In this study, some bioinformatics methods were used to analyze the physicochemical properties, subcellular localization, conserved motif and related phylogenetic tree of *Dof* transcription factors of *C. oleifera*, providing a theoretical basis for further exploration of the function of Dof transcription factors.

In this study, Dof protein sequence information of *C.oleifera* was used to comprehensively analyze Dof family of *C.oleifera*, and 45 *ColDof* genes were identified. Analysis of their domains and motifs showed that all of them contained complete C2-C2 single zinc finger structure, indicating that the Dof transcription factor family was conservative in the process of species evolution.

Studies have shown that the theoretical isoelectric points of ColDof proteins in different plants are basically 5.41 ∼ 6.97, and the number of basic amino acids is generally higher than that of acidic amino acids. However, in this study, the isoelectric points of ColDof proteins in *C.oleifera* were mainly concentrated in 4.89 ∼ 9.65, and most of them were alkaline amino acids, indicating that the isoelectric points of Dof family members of different plants were very different (Supplementary Table 2). According to the prediction analysis of amino acid transmembrane structure, hydrophilicity and phosphorylation sites of Dof family members, it can be concluded that these 45 ColDof proteins were non-transmembrane proteins, and all proteins were hydrophilic proteins. Their protein function was mainly achieved by phosphorylation at the serine site (Table 1). Subcellular localization prediction results showed that *ColDof* genes were all located in the nucleus, and if they were located on the cell membrane, they might be expressed in organelles such as chloroplasts and Golgi apparatus, or in the cytoplasm, indicating that the functions of these genes might also be specific [54]. So far, there were relatively few reports on the existence of signaling peptides in *Dof* gene. A signal peptide was a chain of peptides in a protein molecule that has the ability to transmit signals outside or inside the cell. In soybean *GmDof* genes, the promoter region contains a conserved region that may have a potential signal peptide sequence. However, the prediction of *Dof* gene signal peptide in *C.oleifera* showed no signal peptide.

According to predicted results of secondary structure, 44 ColDof proteins mainly had random coil, and the contents of α-helix, extended chain structure and β-turn are less, and the order of secondary structure component content of each ColDof protein was random coil > α-helix > extended chain > β-turn. Only ColDof12 was dominated by α-helix, which is manifested as α-helix > random coil > extended chain > β-turn (Supplementary Table 4, Fig. 3). The images of the tertiary structure were consistent with the results of the secondary structure prediction. In addition, a total of 10 independent conserved motifs were identified by analyzing 45 ColDof proteins using the online tool MEME. motif1, characterized by a single zinc finger structure (C2-C2), was a core component of Dof protein in *C.oleifera* and is present in 43 ColDof proteins.

The cis-acting elements of promoter region regulate accurate initiation and transcription efficiency of gene transcription by binding with transcription factors, and can identify the core region of transcription activation [55, 56]. *ColDof* gene family contained a large number of cis-acting elements related to photosensitivity, hormonal response, biological and abiotic stress response, which are speculated to play a role in growth and development, stress tolerance and hormone signaling of *C. oleifera*. This was consistent with the study results of Merlino et al. [57] on *Dof* gene family in barley. This research results were also consistent with the study results of Song et al. [58] on *Dof* gene family in *Helianthus annuus*. These results were also consistent with study results of Luo et al. [59] on *Dof* gene family in *Camelina sativa.*

Codons that code for the same amino acid were called synonymous codons, and they were used at different frequencies during translation. This unbalanced codon use phenomenon was called codon use bias [60]. The study of codon preference was conducive to the exploration of genetic evolution, understanding of gene expression characteristics, and providing guidance for molecular breeding. In this study, the codon use preference analysis showed that 45 *ColDof* genes had a slight preference for codon selection. Only AGA has an RSCU greater than 2, indicating a strong preference for this codon among *ColDof* genes. The average content of GC3s and GC was less than 50%, and the high-use codon preference ends with A/U(T), indicating that A/U(T) was used more frequently in the coding sequence codon than G/C. These results were consistent with the results of Wang et al. [61] on the codon preference in chloroplast genome of theaceae.

MiRNA regulated a variety of genes and participates in a variety of biological processes, indicating the complexity of miRNA regulation of target genes [62]. It was found that ath-miR5021 had the largest number of target genes, and most miRNA maturation sequences (5’-3’) were 20 bp in length. As a codominant genetic marker with high polymorphism, good repeatability and strong specificity, SSR played an important role in the analysis of species genetic diversity, the comparison of relatives and the construction of genetic maps [63]. In this study, multiple SSR loci of various types were screened, which could provide data reference for further development of specific SSR markers and genetic diversity analysis of *C. oleifera* [64]. The analysis of SSR sites by IPK online tool software showed that the proportion of single nucleotide repeats was the largest, and the frequency of A/T was also the largest. CT/TC is the main motif of dinucleotide. Except for complex nucleotides, the types of SSR motifs increased with the increasing number of motifs, while the number of SSR loci decreased with the increasing number of motifs.

When plants are exposed to hypothermia stress, the hypothermia receptors in cells can rapidly sense the ambient temperature, and then transmit the information to the nucleus through various transduction pathways [65]. TFs genes that can respond to hypothermia stress in cells begin to express, thereby regulating the expression of downstream related genes, and ultimately affecting the plant response to low temperature [66]. At present, a variety of TFs involved in the regulation of plant hypothermia response have been identified, such as AP2/ERF [67], bHLH [68], MYB [69] and other TFs family members. In this study, 29,912 TFs sequences belonging to 43 TFs families were identified, among which ERF, MYB and Dof were the most abundant TFs families. The *AP2/ERF* family is one of the largest transcription factor families in the plant kingdom, and its members can participate in plant response to low temperature and enhance plant cold resistance by regulating the expression of downstream target genes [70]. Dof family members were widely involved in plant response to low temperature stress, and overexpression of homologous genes encoding Dof can improve the cold tolerance of transgenic plants. Previous studies have shown that grape *VaDof17d* gene played a positive role in grape cold tolerance and may be an important candidate gene for molecular breeding of cold resistance [71]. Therefore, it was speculated that TFs such as *ERF* and *Dof* played an important role in the cold tolerance of *C. oleifera*.

Through collinearity analysis, it was found that there were multiple homologous *Dof* gene pairs between each of two genomes of *C. oleifera*, Yunkang 10, Longjing 43, Shuchazao, Tieguanyin, Biyun, Camellia haploid and Camellia diploid, indicating high collinearity between *C. oleifera* and these genomes. Many plant gene families evolve and expand due to gene replication events, which may also facilitate the formation of new functional genes and species that are better able to withstand harsh environments as plants evolve [72]. Numerous studies have shown that genes generated through fragment replication events may be more likely to be preserved due to subfunctionalization without increasing the likelihood of gene rearrangement [73]. Previous collinearity studies of *Dof* gene families in Tartary buckwheat [74], rose [75], and cotton [76] have shown that segmental repeat events play a dominant role in *Dof* gene expansion. Similarly, no tandem repeat events were observed in *ColDof* genes in *C.oleifera*, and fragment repeats were the primary cause of their amplification, suggesting that some *ColDof* genes may have originated from genetic repeats in *C.oleifera*. However, studies have also shown that tandem repeats and segmentary repeats exist in both *Dof* transcription factors in *Brassica napus* [38] and poplar [77]. Previous studies have shown that phylogenetic tree can provide valuable theoretical basis for function prediction of similar genes in different species, that is, genes clustered in the same group in phylogenetic evolution are relatively conserved in terms of gene structure, protein conserved motif, gene expression pattern, etc. Therefore, genes in the same group may have similar biological functions [78]. The *Dof* gene family of Yunkang No. 10, Longjing No. 43, Shuchazao, Tieguanyin, Biyun, etc. were closely related to *C.oleifera*, because these are all Camellia species of Camellia family. Among them, ColDof11 was most closely related to CSSDof46. It was most closely related to CSSDof31, a member of *Stenophyllum camellia*.

Zhang et al. [79] reported that *CsYABBY10* and *CsYABBY5* genes in tea trees have significant drought and salt tolerance functions. *CoYABBY* gene family in *C. oleifera* genome has significant salt tolerance functions, and *CoYABBY3* gene has the strongest salt tolerance function. So far, no research reports have been found on the salt and drought tolerance of *Dof* gene family in *C. oleifera* genome. However, there are currently many research reports on the salt stress tolerance of *Dof* gene family in plant genomes. Li et al. [80] reported that the silencing of *Dof1.7* gene in the cotton genome significantly reduces the mechanism of cotton’s salt stress response, indicating that *Dof1.7* in cotton genome has a significant salt stress tolerance function. Nan et al. [81] reported that *RchDof9*,* RchDof10*,* RchDof17* and *RchDof20* genes in *Rosa chinensis* genome exhibit significant molecular mechanisms underlying salt stress tolerance responses. Zhou et al. [82] reported that the ClDof29 gene in watermelon has significant salt tolerance. The above indicates that *Dof* gene family in the plant genome has a certain molecular mechanism of salt stress tolerance, which is similar to the results found in this study that *ColDof1*,* ColDof2*,* ColDof14* and *ColDof36* have significant salt tolerance.

Yu et al. [83] reported that most members of *Dof* gene family in the tea plant genome have a molecular mechanism for drought resistance. Sun et al. [84] reported that *BpDof4*,* BpDof11* and *BpDof17* in the Betula platyphylla genome exhibit significant molecular mechanisms of drought stress tolerance. Chen et al. [85] reported that the MdDof54 gene in the apple genome exhibits significant drought resistance. The above research results are similar to the findings of this study, which suggest that *ColDof1*,* ColDof2*,* ColDof5*,* ColDof14*,* ColDof27* and *ColDof36* may be involved in their response to drought stress.

The results of this study provide a reference for further research on the biological functions of *Dof* gene family in *C.oleifera* during its growth and development.

## Conclusion

In this study, we have identified 45 ColDof proteins in *C.oleifera* genome. All the 45 ColDof members are non-transmembrane and non-secretory proteins. The biological function of ColDof proteins was mainly realized by phosphorylation at serine (Ser) site. ColDof genes’ promoter contains a variety of cis-acting element elements, including light response, gibberellin response, abscisic acid response, auxin response and drought induction elements. *ColDof* gene family was most closely related to that of diploid tea tree and *Camellia lanceoleosa*. There were 40 colinear locis between *ColDof* with Dof protein of *Arabidopsis thaliana*. ColDof34, ColDof20, ColDof28, ColDof35, ColDof42 and ColDof26 have the most protein interactions. Moreover, *ColDof1*,* ColDof2*,* ColDof14* and *ColDof36* not only have significant molecular mechanisms for salt stress tolerance, but also significant molecular functions for drought stress tolerance. This study systematically identified the genetic characteristics, protein characteristics, and molecular evolutionary relationships of *Dof* gene family in *C. oleifera* genome, and elucidated the involvement of most *ColDof* genes in the growth and development process of *C. oleifera*, especially in the response to salt stress and drought stress of *C. oleifera*.The results of this study provide a reference for further understanding of the function of *ColDof* genes in *C.oleifera*.

### Electronic supplementary material

Below is the link to the electronic supplementary material.

Supplementary Material 1

Supplementary Material 2

Supplementary Material 3

Supplementary Material 4

Supplementary Material 5

## Data Availability

The genome sequences, protein sequences and gene annotation files of C. oleifera are downloaded in GitHub: https://github.com/Hengfu-Yin/CON_genome_data or Zenodo: https://zenodo.org/record/5768785. *C. oleifera* seed transcriptomics data was downloaded from NCBI (https://www.ncbi.nlm.nih.gov/geo/query/acc.cgi?acc=GSE190644).
