# Supplementary material for: Genome-wide identification and molecular evolution of Dof gene family in Camellia oleifera
Source: BMC Genomics. 2024 Jul 18;25:702. doi: 10.1186/s12864-024-10622-6 (PMC11264790; doi:10.1186/s12864-024-10622-6)
Supplement: Supplementary file 1 — Supplementary Material 1 [file 12864_2024_10622_MOESM1_ESM.docx]

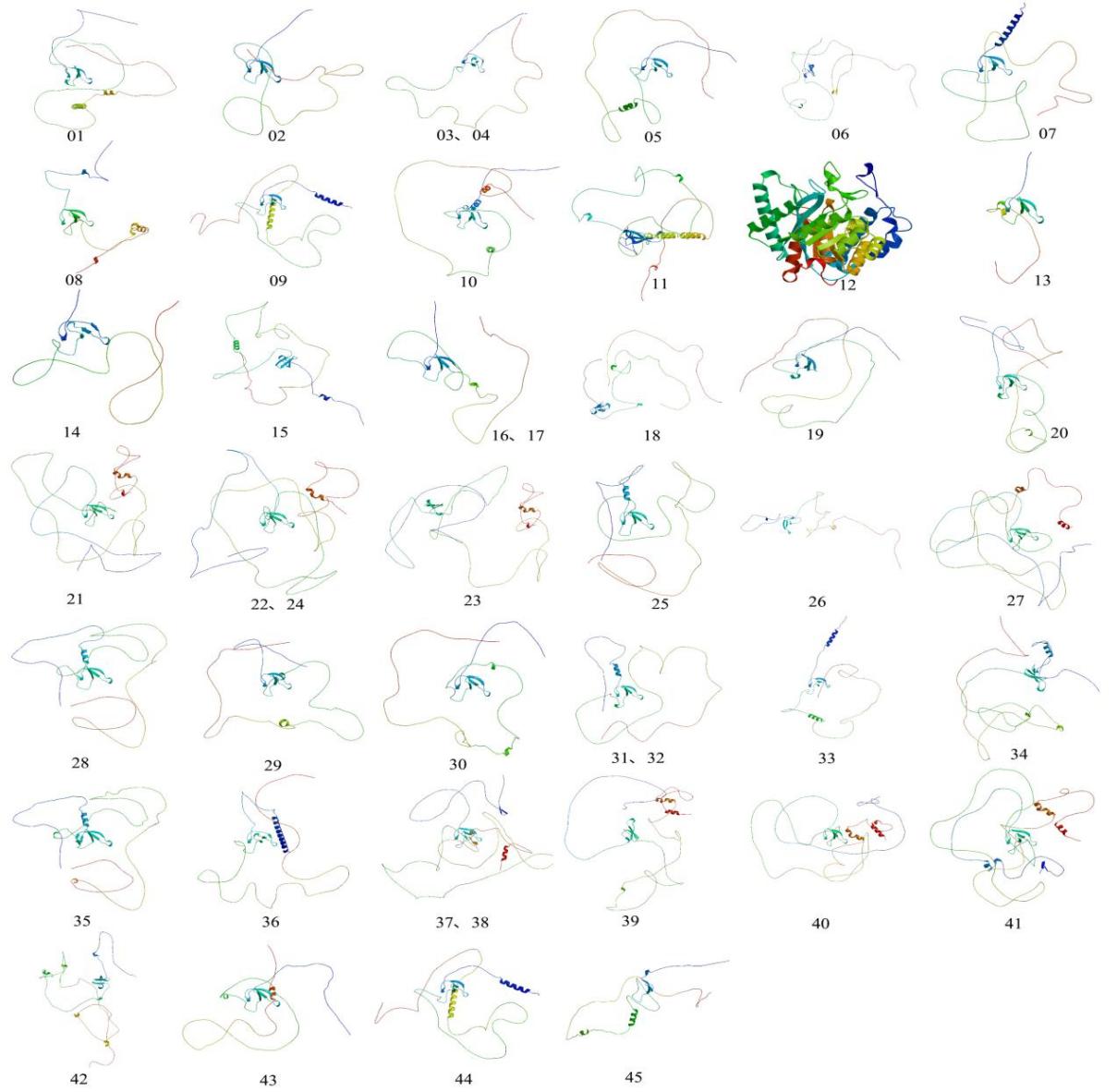


Supplementary Fig. 1 the tertiary structure of Dof proteins in *C.oleifera*

01-ColDof1;02-ColDof2;03-ColDof3;04-ColDof4;05-ColDof5;06-ColDof6;

07-ColDof7;08-ColDof8;09-ColDof9;10-ColDof10;11-ColDof11;12-ColDof12;

13-ColDof13;14-ColDof14;15-ColDof15;16-ColDof16;17-ColDof17;18-ColDof18;

19-ColDof19;20-ColDof20;21-ColDof21;22-ColDof22;23-ColDof23;24-ColDof24;

25-ColDof25;26-ColDof26;27-ColDof27;28-ColDof28;29-ColDof29;30-ColDof30;

31-ColDof31;32-ColDof32;33-ColDof33;34-ColDof34;35-ColDof35;36-ColDof36;

37-ColDof37;38-ColDof38;39-ColDof39;40-ColDof40;41-ColDof41;42-ColDof42;

43-ColDof43;44-ColDof44;45-ColDof45;


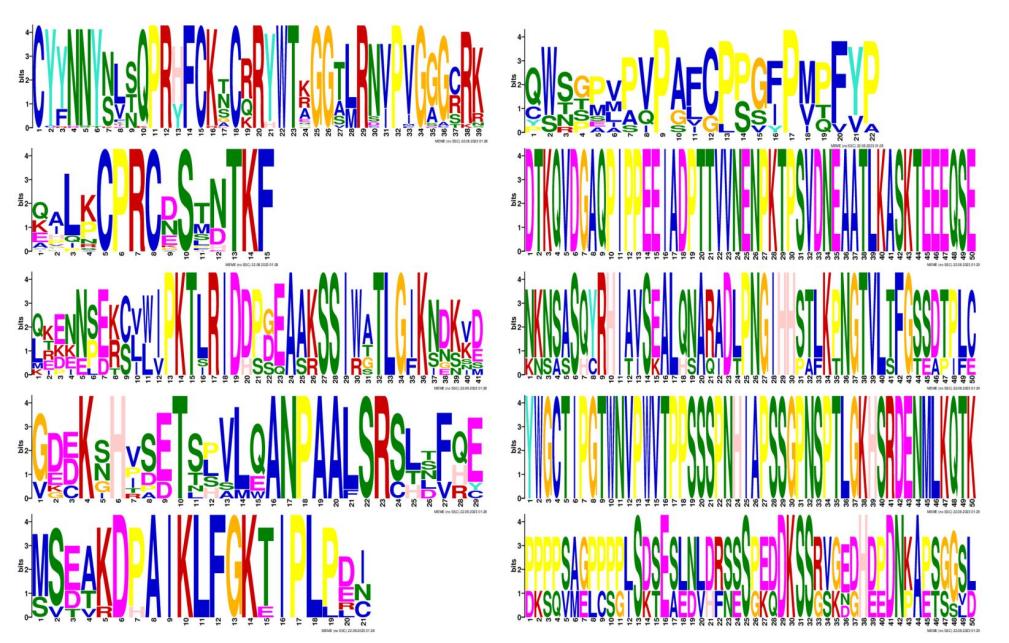


Supplementary Fig. 2 Sequence logo of the conservative motifs


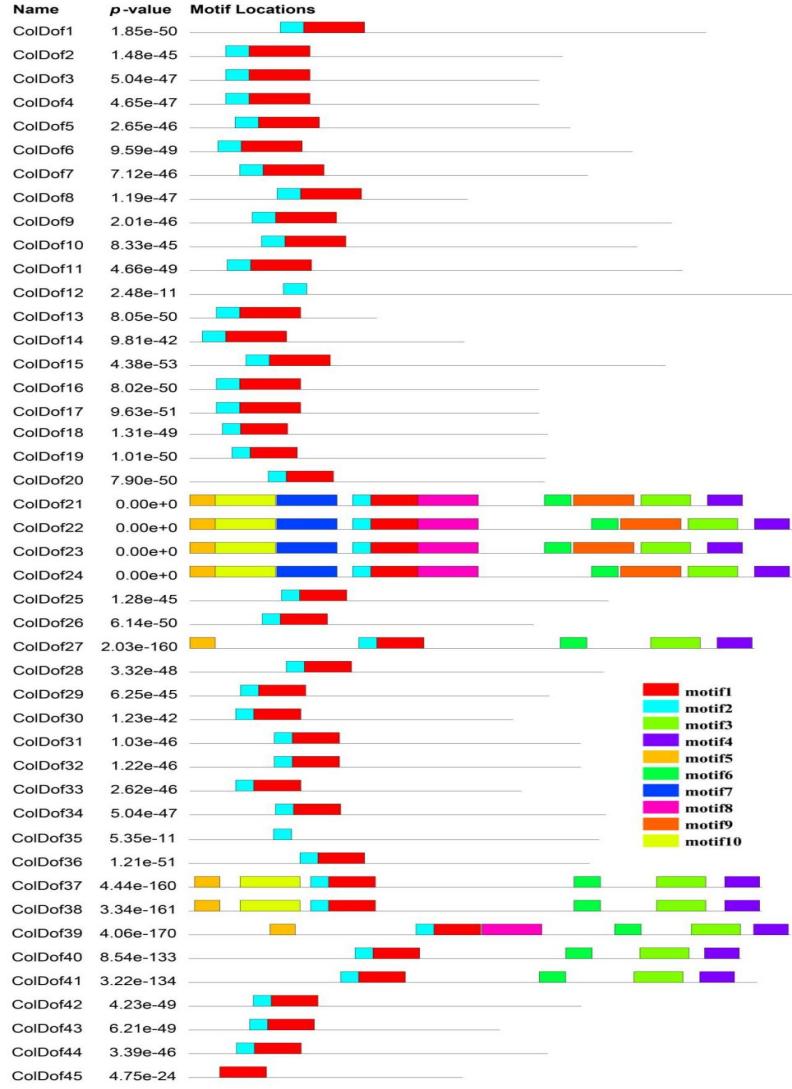


Supplementary Fig 3 conservative motif of ColDof proteins


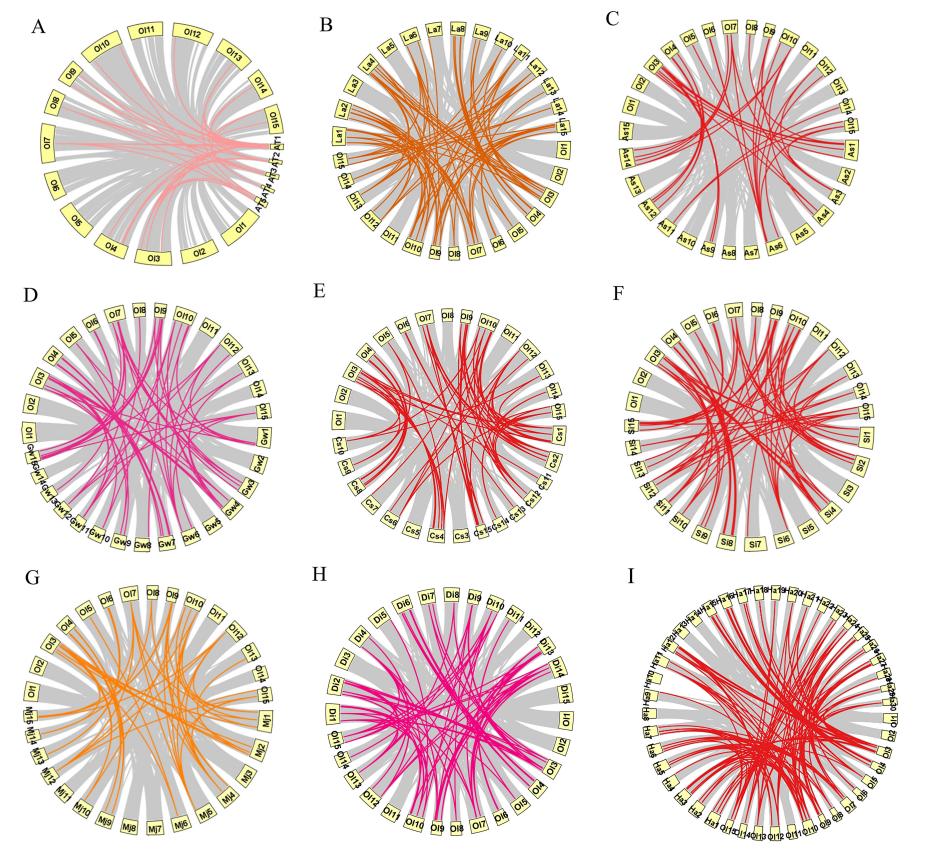


Supplementary Fig.4 Collinearity analysis of *Dof* gene family between *C.oleifera* and other representative species


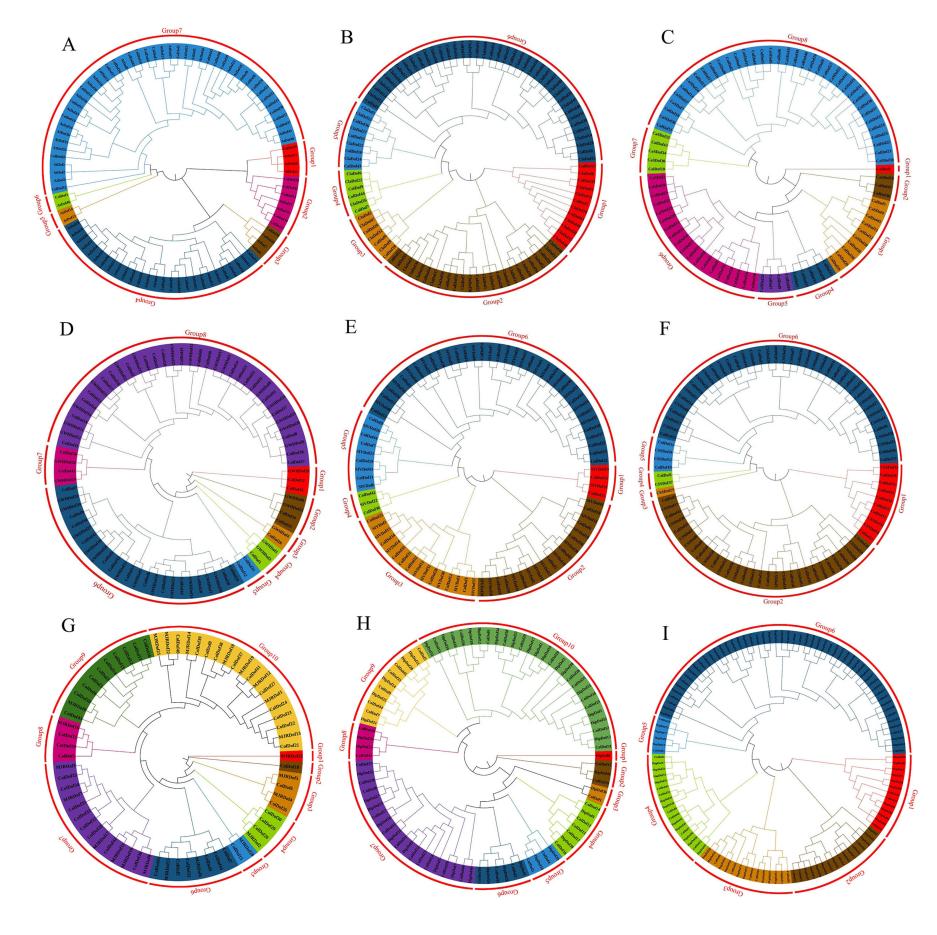


Supplementary Fig.5 Phylogenetic tree of Dof gene family in *Camellia oleifa* and other representative species
