# Supplementary material for: Genome-wide identification and molecular evolution of Dof gene family in Camellia oleifera
Source: BMC Genomics. 2024 Jul 18;25:702. doi: 10.1186/s12864-024-10622-6 (PMC11264790; doi:10.1186/s12864-024-10622-6)
Supplement: Supplementary file 2 — Supplementary Material 2 [file 12864_2024_10622_MOESM2_ESM.docx]

Supplementary Table 2 Basic information of Dof family genes in *C.oleifera*

| Gene name | Gene ID | Location | Sequence length/bp |
| --- | --- | --- | --- |
| ColDof1 | augustus_masked-HiC_scaffold_10-processed-gene-1834.19-mRNA-1 | Chr10：183452222-183453217 | 995 |
| ColDof2 | augustus_masked-HiC_scaffold_10-processed-gene-1852.7-mRNA-1 | Chr10：185249664-185250383 | 719 |
| ColDof3 | augustus_masked-HiC_scaffold_3-processed-gene-149.24-mRNA-1 | Chr3：14927991-14928665 | 674 |
| ColDof4 | augustus_masked-HiC_scaffold_3-processed-gene-152.40-mRNA-1 | Chr3：15209895-15210569 | 674 |
| ColDof5 | augustus_masked-HiC_scaffold_3-processed-gene-536.31-mRNA-1 | Chr3：53648982-53649716 | 734 |
| ColDof6 | augustus_masked-HiC_scaffold_3-processed-gene-831.21-mRNA-1 | Chr3：83161168-83162022 | 854 |
| ColDof7 | augustus_masked-HiC_scaffold_4-processed-gene-1269.10-mRNA-1 | Chr4：126943675-126944442 | 767 |
| ColDof8 | augustus_masked-HiC_scaffold_4-processed-gene-332.15-mRNA-1 | Chr4：33210151-33210687 | 536 |
| ColDof9 | augustus_masked-HiC_scaffold_4-processed-gene-399.13-mRNA-1 | Chr4：39934572-39935501 | 929 |
| ColDof10 | augustus_masked-HiC_scaffold_4-processed-gene-429.20-mRNA-1 | Chr4：42954848-42955711 | 863 |
| ColDof11 | augustus_masked-HiC_scaffold_7-processed-gene-1069.9-mRNA-1 | Chr7：106915237-106916187 | 950 |
| ColDof12 | augustus_masked-HiC_scaffold_7-processed-gene-1130.2-mRNA-1 | Chr7：113062640-113063800 | 1160 |
| ColDof13 | augustus_masked-HiC_scaffold_7-processed-gene-1833.2-mRNA-1 | Chr7：183314218-183314580 | 362 |
| ColDof14 | augustus_masked-HiC_scaffold_7-processed-gene-491.17-mRNA-1 | Chr7：49151737-49152267 | 530 |
| ColDof15 | augustus_masked-HiC_scaffold_8-processed-gene-563.41-mRNA-1 | Chr8：56290722-56293284 | 2562 |
| ColDof16 | augustus_masked-HiC_scaffold_9-processed-gene-135.26-mRNA-1 | Chr9：13558843-13559517 | 674 |
| ColDof17 | augustus_masked-HiC_scaffold_9-processed-gene-136.45-mRNA-1 | Chr9：13671891-13672565 | 674 |
| ColDof18 | augustus_masked-HiC_scaffold_9-processed-gene-727.23-mRNA-1 | Chr9：72772025-72772918 | 893 |
| ColDof19 | augustus_masked-HiC_scaffold_9-processed-gene-984.22-mRNA-1 | Chr9：98482122-98483009 | 887 |
| ColDof20 | maker-HiC_scaffold_10-snap-gene-1047.27-mRNA-1 | Chr10：104726037-104732862 | 6825 |
| ColDof21 | maker-HiC_scaffold_10-snap-gene-1980.23-mRNA-1 | Chr10：197999273-198004790 | 5517 |
| ColDof22 | maker-HiC_scaffold_10-snap-gene-1980.23-mRNA-2 | Chr10：197999273-198003600 | 4327 |
| ColDof23 | maker-HiC_scaffold_10-snap-gene-1981.0-mRNA-1 | Chr10：198104537-198110273 | 5736 |
| ColDof24 | maker-HiC_scaffold_10-snap-gene-1981.0-mRNA-2 | Chr10：198104537-198108844 | 4307 |
| ColDof25 | maker-HiC_scaffold_10-snap-gene-293.0-mRNA-2 | Chr10：29330838-29332704 | 1866 |
| ColDof26 | maker-HiC_scaffold_12-snap-gene-1705.3-mRNA-1 | Chr12：170549098-170550046 | 948 |
| ColDof27 | maker-HiC_scaffold_12-snap-gene-172.48-mRNA-1 | Chr12：17259909-17262716 | 2807 |
| ColDof28 | maker-HiC_scaffold_13-snap-gene-1239.0-mRNA-1 | Chr13：123891132-123892882 | 1750 |
| ColDof29 | maker-HiC_scaffold_14-snap-gene-560.3-mRNA-1 | Chr14：56028361-56030391 | 2030 |
| ColDof30 | maker-HiC_scaffold_15-snap-gene-22.4-mRNA-1 | Chr15：2236123-2238151 | 2028 |
| ColDof31 | maker-HiC_scaffold_3-snap-gene-1391.31-mRNA-1 | Chr3：139161844-139163559 | 1715 |
| ColDof32 | maker-HiC_scaffold_3-snap-gene-1405.12-mRNA-1 | Chr3：140539325-140541041 | 1716 |
| ColDof33 | maker-HiC_scaffold_3-snap-gene-1473.0-mRNA-1 | Chr3：147380094-147381925 | 1831 |
| ColDof34 | maker-HiC_scaffold_3-snap-gene-1731.30-mRNA-1 | Chr3：173130122-173131635 | 1513 |
| ColDof35 | maker-HiC_scaffold_3-snap-gene-1731.31-mRNA-1 | Chr3：173156928-173158313 | 1385 |
| ColDof36 | maker-HiC_scaffold_4-snap-gene-395.0-mRNA-2 | Chr4：39499717-39501813 | 2096 |
| ColDof37 | maker-HiC_scaffold_6-snap-gene-540.2-mRNA-1 | Chr6：54041677-54044884 | 3207 |
| ColDof38 | maker-HiC_scaffold_6-snap-gene-618.15-mRNA-1 | Chr6：61810600-61813806 | 3206 |
| ColDof39 | maker-HiC_scaffold_7-snap-gene-1017.1-mRNA-1 | Chr7：101701252-101704348 | 3096 |
| ColDof40 | maker-HiC_scaffold_9-snap-gene-887.6-mRNA-1 | Chr9：88712637-88715958 | 3321 |
| ColDof41 | snap_masked-HiC_scaffold_15-processed-gene-796.3-mRNA-1 | Chr15：79600112-79603784 | 3672 |
| ColDof42 | snap_masked-HiC_scaffold_4-processed-gene-1268.16-mRNA-1 | Chr4：126803327-126805563 | 2236 |
| ColDof43 | snap_masked-HiC_scaffold_4-processed-gene-1325.5-mRNA-1 | Chr4：132527339-132528121 | 782 |
| ColDof44 | snap_masked-HiC_scaffold_4-processed-gene-396.12-mRNA-1 | Chr4：39630205-39631134 | 929 |
| ColDof45 | snap_masked-HiC_scaffold_5-processed-gene-982.16-mRNA-1 | Chr5：98279904-98280648 | 744 |

Supplementary Table.3 Physicochemical properties of Dof proteins from *C.oleifera*

| Protein | Amino acid number/aa | MW/kDa | pI | Instability index | Aliphatic index | Average of  hydropathicity  (GEAVY) | Asp+Glu | Arg+Lys | subcellular localization |
| --- | --- | --- | --- | --- | --- | --- | --- | --- | --- |
| ColDof01 | 331 | 35.50838 | 9.21 | 61.45 | 67.49 | -0.586 | 16 | 24 | Nuclear |
| ColDof02 | 239 | 25.08093 | 6.69 | 44.34 | 58.79 | -0.315 | 18 | 17 | Nuclear |
| ColDof03 | 224 | 23.49802 | 7.58 | 52.07 | 51.29 | -0.505 | 18 | 19 | Nuclear |
| ColDof04 | 224 | 23.51402 | 7.58 | 52.93 | 50.85 | -0.517 | 18 | 19 | Nuclear |
| ColDof05 | 244 | 26.90586 | 4.89 | 47.50 | 63.52 | -0.497 | 30 | 20 | Nuclear |
| ColDof06 | 284 | 31.90584 | 7.19 | 38.74 | 51.80 | -0.739 | 28 | 28 | Nuclear |
| ColDof07 | 255 | 28.20715 | 9.65 | 59.65 | 44.75 | -1.022 | 10 | 23 | Nuclear |
| ColDof08 | 178 | 19.96432 | 9.10 | 54.86 | 47.13 | -0.816 | 18 | 25 | Nuclear |
| ColDof09 | 309 | 34.36650 | 9.26 | 74.97 | 37.31 | -1.191 | 13 | 21 | Nuclear |
| ColDof10 | 287 | 31.76106 | 7.07 | 55.72 | 55.05 | -0.713 | 27 | 27 | Nuclear |
| ColDof11 | 316 | 34.59593 | 8.41 | 47.21 | 50.03 | -0.791 | 24 | 27 | Nuclear |
| ColDof12 | 386 | 42.27944 | 6.48 | 46.03 | 84.43 | -0.262 | 46 | 44 | Nuclear |
| ColDof13 | 120 | 13.45278 | 9.51 | 63.26 | 30.17 | -1.155 | 8 | 16 | Nuclear |
| ColDof14 | 176 | 19.36427 | 9.22 | 54.61 | 41.53 | -0.964 | 13 | 19 | Nuclear |
| ColDof15 | 305 | 33.04063 | 7.12 | 46.18 | 52.39 | -0.702 | 26 | 26 | Nuclear |
| ColDof16 | 224 | 24.28555 | 8.61 | 48.26 | 46.21 | -0.821 | 16 | 19 | Nuclear |
| ColDof17 | 224 | 24.26955 | 8.61 | 48.59 | 47.10 | -0.808 | 16 | 19 | Nuclear |
| ColDof18 | 297 | 32.34912 | 7.61 | 42.88 | 47.91 | -0.718 | 26 | 27 | Nuclear |
| ColDof19 | 295 | 32.22414 | 6.52 | 50.79 | 50.34 | -0.759 | 27 | 24 | Nuclear |
| ColDof20 | 294 | 32.17395 | 9.15 | 39.45 | 62.69 | -0.603 | 23 | 31 | Nuclear |
| ColDof21 | 459 | 49.42782 | 5.97 | 53.51 | 53.14 | -0.823 | 52 | 46 | Nuclear |
| ColDof22 | 499 | 53.86484 | 5.93 | 52.92 | 54.35 | -0.794 | 57 | 50 | Nuclear |
| ColDof23 | 459 | 49.48802 | 5.97 | 54.47 | 53.14 | -0.816 | 52 | 46 | Nuclear |
| ColDof24 | 499 | 53.86500 | 5.93 | 53.96 | 54.35 | -0.780 | 57 | 50 | Nuclear |
| ColDof25 | 638 | 70.04240 | 7.56 | 50.36 | 56.57 | -0.591 | 55 | 56 | Nuclear |
| ColDof26 | 285 | 32.36723 | 5.98 | 43.98 | 53.02 | -0.754 | 33 | 28 | Nuclear |
| ColDof27 | 468 | 51.62719 | 6.54 | 49.97 | 57.50 | -0.820 | 52 | 49 | Nuclear |
| ColDof28 | 343 | 35.78371 | 9.30 | 61.03 | 48.95 | -0.601 | 17 | 25 | Nuclear |
| ColDof29 | 298 | 32.37919 | 8.07 | 57.23 | 64.73 | -0.508 | 25 | 27 | Nuclear |
| ColDof30 | 268 | 29.64011 | 8.38 | 62.48 | 55.60 | -0.715 | 24 | 27 | Nuclear |
| ColDof31 | 324 | 34.26624 | 9.23 | 54.44 | 56.57 | -0.538 | 17 | 26 | Nuclear |
| ColDof32 | 324 | 34.25925 | 9.23 | 54.20 | 56.88 | -0.530 | 17 | 26 | Nuclear |
| ColDof33 | 275 | 30.79631 | 7.25 | 47.28 | 50.69 | -0.845 | 25 | 25 | Nuclear |
| ColDof34 | 345 | 36.33641 | 9.02 | 54.11 | 57.65 | -0.543 | 18 | 25 | Nuclear |
| CanDof35 | 343 | 36.36050 | 7.14 | 59.12 | 63.12 | -0.512 | 23 | 23 | Nuclear |
| CanDof36 | 335 | 37.30580 | 9.42 | 63.26 | 68.66 | -0.568 | 21 | 32 | Nuclear |
| CanDof37 | 479 | 52.13115 | 6.33 | 45.45 | 54.30 | -0.676 | 54 | 50 | Nuclear |
| CanDof38 | 479 | 52.17127 | 6.64 | 43.72 | 54.91 | -0.671 | 53 | 51 | Nuclear |
| CanDof39 | 503 | 55.62664 | 7.81 | 49.52 | 57.83 | -0.783 | 67 | 69 | Nuclear |
| CanDof40 | 462 | 50.74663 | 5.73 | 50.84 | 57.66 | -0.825 | 60 | 54 | Nuclear |
| CanDof41 | 475 | 52.67512 | 6.61 | 57.89 | 62.40 | -0.704 | 55 | 53 | Nuclear |
| CanDof42 | 328 | 36.36925 | 8.63 | 52.96 | 58.20 | -0.754 | 23 | 27 | Nuclear |
| CanDof43 | 260 | 28.29924 | 7.62 | 59.40 | 46.92 | -0.730 | 23 | 24 | Nuclear |
| CanDof44 | 300 | 33.39246 | 9.39 | 74.81 | 37.13 | -1.189 | 12 | 21 | Nuclear |
| CanDof45 | 229 | 25.91604 | 4.96 | 54.80 | 67.21 | -0.656 | 30 | 25 | Nuclear |

Supplementary Table.4 Hydrophilicity / hydrophobicity analysis of Dof proteins in *C.oleifera*

| Protein name | Maximum hydrophobicity | | | Maximum hydrophilicity | | |
| --- | --- | --- | --- | --- | --- | --- |
|  | Position | amibo  teminal | Value | Position | amibo  teminal | Value |
| ColDof01 | 9 | G | 1.789 | 244、255 | H、L | -3.433 |
| ColDof02 | 155 | G | 1.956 | 11 | T | -3.100 |
| ColDof03 | 152 | G | 1.644 | 80 | N | -3.011 |
| ColDof04 | 152 | G | 1.644 | 80 | N | -3.011 |
| ColDof05 | 129 | F | 1.511 | 86、87 | R、A | -2.133 |
| ColDof06 | 176 | G | 0.978 | 15 | P | -2.778 |
| ColDof07 | 132 | S | 1.767 | 28 | H | -3.478 |
| ColDof08 | 140 | D | 1.589 | 45 | H | -3.089 |
| ColDof09 | 161 | S | 1.544 | 17 | H | -3.522 |
| ColDof10 | 248 | T | 1.100 | 69 | E | -2.856 |
| ColDof11 | 203 | N | 1.611 | 21 | H | -3.267 |
| ColDof12 | 191 | L | 2.078 | 214 | N | -2.944 |
| ColDof13 | 113 | A | 1.700 | 74 | K | -3.200 |
| ColDof14 | 132 | G | 1.033 | 65 | T | -2.589 |
| ColDof15 | 288 | L | 1.556 | 30 | K | -3.278 |
| ColDof16 | 113 | A | 1.700 | 74 | K | -3.200 |
| ColDof17 | 113 | A | 1.700 | 74 | K | -3.200 |
| ColDof18 | 180 | G | 1.044 | 240 | H | -3.089 |
| ColDof19 | 134 | A | 1.544 | 32 | Q | -3.367 |
| ColDof20 | 27 | S | 3.133 | 119 | K | -2.522 |
| ColDof21 | 452 | R | 1.322 | 375 | Q | -3.400 |
| ColDof22 | 271 | V | 1.900 | 414 | Q | -3.400 |
| ColDof23 | 452 | R | 1.322 | 375 | Q | -3.333 |
| ColDof24 | 271 | V | 1.900 | 414 | Q | -3.333 |
| ColDof25 | 6 | V | 1.633 | 133、134 | K、S | -3.667 |
| ColDof26 | 6、7 | D、I | 1.144 | 57 | P | -2.778 |
| ColDof27 | 210 | Q | 1.678 | 45、46 | D、D | -3.400 |
| ColDof28 | 6 | I | 1.311 | 137 | R | -2.978 |
| ColDof29 | 294 | L | 1.656 | 271 | E | -2.967 |
| ColDof30 | 173 | T | 1.233 | 35 | P | -2.811 |
| ColDof31 | 277 | V | 1.933 | 127 | K | -2.667 |
| ColDof32 | 277 | V | 1.933 | 127 | K | -2.667 |
| ColDof33 | 162 | F | 1.178 | 153 | H | -3.367 |
| ColDof34 | 166 | S | 1.678 | 18、19 | Q、Q | -3.000 |
| ColDof35 | 164 | S | 1.678 | 128 | E | -3.489 |
| ColDof36 | 31 | L | 2.900 | 40 | Q | -2.611 |
| ColDof37 | 331 | P | 1.600 | 433 | T | -3.089 |
| ColDof38 | 331 | P | 1.600 | 433 | T | -3.089 |
| ColDof39 | 40 | F | 2.389 | 20 | R | -4.056 |
| ColDof40 | 324 | P | 1.911 | 122 | N | -3.544 |
| ColDof41 | 465、466 | V、I | 1.500 | 32、33 | D、D | -3.500 |
| ColDof42 | 242 | L | 1.878 | 49、50、51 | Q、Q、Q | -3.500 |
| ColDof43 | 189 | L | 1.189 | 74 | K | -2.900 |
| ColDof44 | 161 | S | 1.544 | 17 | H | -3.522 |
| ColDof45 | 122 | T | 1.511 | 127、129 | R、Q | -3.722 |

Supplementary Table 5 Secondary structure analysis of Dof proteins from *C.oleifera*

| Protein | Alpha helix | % | Extended strand | % | Beta turn | % | Random coil | % |
| --- | --- | --- | --- | --- | --- | --- | --- | --- |
| ColDof01 | 46 | 13.90 | 44 | 13.29 | 8 | 2.42 | 233 | 70.39 |
| ColDof02 | 20 | 8.37 | 46 | 19.25 | 12 | 5.02 | 161 | 67.36 |
| ColDof03 | 13 | 5.80 | 32 | 14.29 | 5 | 2.23 | 174 | 77.68 |
| ColDof04 | 13 | 5.80 | 32 | 14.29 | 5 | 2.23 | 174 | 77.68 |
| ColDof05 | 66 | 27.05 | 21 | 8.61 | 7 | 2.87 | 150 | 61.48 |
| ColDof06 | 30 | 10.56 | 51 | 17.96 | 6 | 2.11 | 197 | 69.37 |
| ColDof07 | 20 | 7.84 | 30 | 11.76 | 6 | 2.35 | 199 | 78.04 |
| ColDof08 | 33 | 18.54 | 30 | 16.85 | 12 | 6.74 | 103 | 57.87 |
| ColDof09 | 51 | 16.50 | 28 | 9.06 | 15 | 4.85 | 215 | 69.58 |
| ColDof10 | 27 | 9.41 | 30 | 10.45 | 6 | 2.09 | 224 | 78.05 |
| ColDof11 | 42 | 13.29 | 43 | 13.61 | 18 | 5.70 | 213 | 67.41 |
| ColDof12 | 183 | 47.41 | 51 | 13.21 | 25 | 6.48 | 127 | 32.90 |
| ColDof13 | 10 | 8.33 | 17 | 14.17 | 10 | 8.33 | 83 | 69.17 |
| ColDof14 | 17 | 9.66 | 22 | 12.50 | 9 | 5.11 | 128 | 72.73 |
| ColDof15 | 63 | 20.66 | 45 | 14.75 | 6 | 1.97 | 191 | 62.62 |
| ColDof16 | 15 | 6.70 | 23 | 10.27 | 10 | 4.46 | 176 | 78.57 |
| ColDof17 | 20 | 8.93 | 23 | 10.27 | 9 | 4.02 | 172 | 76.79 |
| ColDof18 | 35 | 11.78 | 50 | 16.84 | 10 | 3.37 | 202 | 68.01 |
| ColDof19 | 42 | 14.42 | 46 | 15.59 | 13 | 4.41 | 194 | 65.76 |
| ColDof20 | 13 | 4.42 | 56 | 19.05 | 6 | 2.04 | 219 | 74.49 |
| ColDof21 | 52 | 11.33 | 40 | 8.71 | 10 | 2.18 | 357 | 77.78 |
| ColDof22 | 58 | 11.62 | 43 | 8.62 | 8 | 1.60 | 390 | 78.16 |
| ColDof23 | 56 | 12.20 | 33 | 7.19 | 10 | 2.18 | 360 | 78.43 |
| ColDof24 | 61 | 12.22 | 40 | 8.02 | 8 | 1.60 | 390 | 78.16 |
| ColDof25 | 33 | 9.51 | 41 | 11.82 | 8 | 2.31 | 265 | 76.37 |
| ColDof26 | 33 | 11.58 | 67 | 23.51 | 15 | 5.26 | 170 | 59.65 |
| ColDof27 | 55 | 11.75 | 40 | 8.55 | 7 | 1.50 | 366 | 78.21 |
| ColDof28 | 30 | 8.75 | 29 | 8.45 | 11 | 3.21 | 273 | 79.59 |
| ColDof29 | 23 | 7.72 | 44 | 14.77 | 6 | 2.01 | 225 | 75.50 |
| ColDof30 | 16 | 5.97 | 33 | 12.31 | 3 | 1.10 | 216 | 80.60 |
| CatDof31 | 37 | 11.42 | 44 | 13.58 | 16 | 4.94 | 227 | 70.06 |
| CatDof32 | 33 | 10.19 | 48 | 14.81 | 21 | 6.48 | 222 | 68.52 |
| CatDof33 | 46 | 16.73 | 30 | 10.91 | 7 | 2.55 | 192 | 69.82 |
| CatDof34 | 32 | 9.28 | 29 | 8.14 | 9 | 2.61 | 275 | 79.71 |
| CatDof35 | 37 | 10.79 | 26 | 7.58 | 6 | 1.75 | 274 | 79.88 |
| CatDof36 | 49 | 14.63 | 30 | 8.96 | 4 | 1.19 | 252 | 75.22 |
| CatDof37 | 63 | 13.15 | 37 | 7.72 | 9 | 1.88 | 370 | 77.24 |
| CatDof38 | 64 | 13.36 | 39 | 8.14 | 14 | 2.92 | 362 | 75.57 |
| CatDof39 | 63 | 12.52 | 51 | 10.14 | 10 | 1.99 | 379 | 75.35 |
| CatDof40 | 67 | 14.50 | 37 | 8.01 | 9 | 1.95 | 349 | 75.54 |
| CatDof41 | 61 | 12.84 | 46 | 9.68 | 11 | 2.32 | 357 | 75.16 |
| CatDof42 | 36 | 10.98 | 36 | 10.98 | 13 | 3.96 | 243 | 74.09 |
| CatDof43 | 17 | 6.54 | 20 | 7.69 | 3 | 1.15 | 220 | 84.62 |
| CatDof44 | 46 | 15.33 | 28 | 9.33 | 14 | 4.67 | 212 | 70.67 |
| CatDof45 | 59 | 25.76 | 21 | 9.17 | 4 | 1.75 | 145 | 63.32 |

Supplementary Table 6 Predicted SSR loci in *ColDolf* genes

| Gene name | SSR type | SSR type | SSR length | start position | end position |
| --- | --- | --- | --- | --- | --- |
| ColDof8 | p3 | (ACA)6 | 18 | 72 | 89 |
| ColDof9 | p3 | (GGT)5 | 15 | 873 | 887 |
| ColDof10 | p3 | (CCA)5 | 15 | 317 | 331 |
| ColDof15 | p1 | (A)15 | 15 | 295 | 309 |
| ColDof15 | p1 | (T)17 | 17 | 429 | 445 |
| ColDof15 | p1 | (A)10 | 10 | 797 | 806 |
| ColDof15 | p3 | (ATC)7 | 21 | 1723 | 1743 |
| ColDof16 | p3 | (GTG)7 | 21 | 590 | 610 |
| ColDof17 | p3 | (GTG)7 | 21 | 590 | 610 |
| ColDof19 | p3 | (GCG)6 | 18 | 479 | 496 |
| ColDof19 | p3 | (TGG)6 | 18 | 794 | 811 |
| ColDof20 | p1 | (A)13 | 13 | 1087 | 1099 |
| ColDof20 | c | (T)15gaattgtttgaactttttttttgaactttctgaatttttttcgggacgacccagtgatcgaccggttaggcaaaa(T)22 | 112 | 3891 | 4002 |
| ColDof20 | c | (T)11acaaattttgcctaaccaaccgttccaccggttgaccggaatagtgaa(T)10 | 69 | 4386 | 4454 |
| ColDof20 | p2 | (CT)27 | 54 | 4994 | 5047 |
| ColDof20 | p1 | (T)12 | 12 | 5350 | 5361 |
| ColDof20 | c | (T)15ctataatcttttgtttttctttttggtgtgtgttgttctatcctt(TA)10 | 80 | 6573 | 6652 |
| ColDof21 | p2 | (TC)17 | 34 | 119 | 152 |
| ColDof21 | p1 | (C)32 | 32 | 1189 | 1220 |
| ColDof21 | p1 | (T)11 | 11 | 4815 | 4825 |
| ColDof21 | p1 | (T)11 | 11 | 5473 | 5483 |
| ColDof22 | p2 | (TC)17 | 34 | 119 | 152 |
| ColDof22 | p1 | (C)32 | 32 | 1189 | 1220 |
| ColDof23 | p2 | (TC)15 | 30 | 114 | 143 |
| ColDof23 | p1 | (C)35 | 35 | 1171 | 1205 |
| ColDof23 | p1 | (T)14 | 14 | 4794 | 4807 |
| ColDof23 | p1 | (T)10 | 10 | 5693 | 5702 |
| ColDof24 | p2 | (TC)15 | 30 | 114 | 143 |
| ColDof24 | p1 | (C)35 | 35 | 1171 | 1205 |
| ColDof25 | p2 | (CT)7 | 14 | 12 | 25 |
| ColDof25 | p3 | (TGG)6 | 18 | 959 | 976 |
| ColDof27 | p2 | (AG)7 | 14 | 1 | 14 |
| ColDof27 | p1 | (T)15 | 15 | 410 | 424 |
| ColDof27 | p1 | (A)17 | 17 | 735 | 751 |
| ColDof28 | p2 | (TC)6 | 12 | 1 | 12 |
| ColDof28 | p1 | (A)14 | 14 | 232 | 245 |
| ColDof28 | p1 | (T)13 | 13 | 388 | 400 |
| ColDof29 | c | (C)25(A)20 | 45 | 459 | 503 |
| ColDof30 | p3 | (ATA)6 | 18 | 41 | 58 |
| ColDof30 | p3 | (TGA)6 | 18 | 996 | 1013 |
| ColDof31 | p3 | (ATT)5 | 15 | 342 | 356 |
| ColDof31 | p1 | (A)10 | 10 | 1214 | 1223 |
| ColDof32 | p3 | (ATT)5 | 15 | 342 | 356 |
| ColDof32 | p1 | (A)12 | 12 | 1214 | 1225 |
| ColDof33 | p2 | (TC)6 | 12 | 1 | 12 |
| ColDof33 | p1 | (A)14 | 14 | 303 | 316 |
| ColDof33 | p2 | (TA)8 | 16 | 633 | 648 |
| ColDof34 | p3 | (ATC)5 | 15 | 916 | 930 |
| ColDof35 | p3 | (ATC)5 | 15 | 914 | 928 |
| ColDof36 | p2 | (CT)19 | 38 | 201 | 238 |
| ColDof36 | p1 | (T)14 | 14 | 1413 | 1426 |
| ColDof36 | p3 | (TCA)6 | 18 | 1679 | 1696 |
| ColDof37 | p1 | (A)11 | 11 | 580 | 590 |
| ColDof37 | p2 | (TG)7 | 14 | 979 | 992 |
| ColDof38 | p1 | (T)10 | 10 | 2619 | 2628 |
| ColDof39 | c | (GA)13atcaccccagaaaacccattctgattcaaacatcgtttcttgttttgtttttatccattttctcggccactttcccgcgttttccggggaaaaagggaag(AAAGA)5 | 151 | 154 | 304 |
| ColDof39 | p2 | (AT)7 | 14 | 530 | 543 |
| ColDof39 | p1 | (T)18 | 18 | 3080 | 3097 |
| ColDof40 | p1 | (T)12 | 12 | 89 | 100 |
| ColDof40 | p1 | (T)10 | 10 | 1245 | 1254 |
| ColDof41 | p3 | (ATG)5 | 15 | 86 | 100 |
| ColDof41 | p1 | (T)16 | 16 | 685 | 700 |
| ColDof41 | p1 | (A)16 | 16 | 969 | 984 |
| ColDof41 | p1 | (A)13 | 13 | 1216 | 1228 |
| ColDof42 | p2 | (TC)16 | 32 | 178 | 209 |
| ColDof42 | p3 | (CAA)7 | 21 | 427 | 447 |
| ColDof43 | p3 | (CCT)5 | 15 | 94 | 108 |

Supplementary Table 8. Base compositions and related codon parameters of Dof family members in *C.oleifera*

| gene | T3s | C3s | A3s | G3s | CAI | CBI | Fop | ENc | GC3s | GC | L_sym | L_aa | Aromo |
| --- | --- | --- | --- | --- | --- | --- | --- | --- | --- | --- | --- | --- | --- |
| ColDof1 | 0.30 | 0.31 | 0.23 | 0.31 | 0.14 | 0.03 | 0.40 | 52.98 | 0.53 | 0.50 | 297 | 311 | 0.06 |
| ColDof2 | 0.24 | 0.41 | 0.27 | 0.25 | 0.20 | 0.08 | 0.46 | 53.09 | 0.56 | 0.58 | 232 | 238 | 0.05 |
| ColDof3 | 0.27 | 0.39 | 0.20 | 0.33 | 0.25 | 0.12 | 0.50 | 53.91 | 0.60 | 0.58 | 218 | 224 | 0.08 |
| ColDof4 | 0.25 | 0.39 | 0.27 | 0.23 | 0.17 | 0.07 | 0.45 | 47.54 | 0.54 | 0.58 | 215 | 222 | 0.06 |
| ColDof5 | 0.42 | 0.29 | 0.25 | 0.29 | 0.21 | -0.07 | 0.39 | 49.42 | 0.45 | 0.48 | 236 | 244 | 0.09 |
| ColDof6 | 0.42 | 0.24 | 0.34 | 0.28 | 0.16 | -0.18 | 0.33 | 52.54 | 0.40 | 0.44 | 266 | 284 | 0.09 |
| ColDof7 | 0.37 | 0.23 | 0.23 | 0.30 | 0.15 | 0.02 | 0.38 | 47.53 | 0.46 | 0.49 | 232 | 246 | 0.07 |
| ColDof8 | 0.35 | 0.36 | 0.33 | 0.27 | 0.23 | -0.06 | 0.41 | 61.00 | 0.48 | 0.51 | 174 | 178 | 0.09 |
| ColDof9 | 0.29 | 0.21 | 0.25 | 0.36 | 0.14 | 0.06 | 0.39 | 52.35 | 0.50 | 0.53 | 274 | 299 | 0.07 |
| ColDof10 | 0.44 | 0.31 | 0.23 | 0.26 | 0.24 | 0.01 | 0.44 | 52.59 | 0.45 | 0.47 | 277 | 287 | 0.09 |
| ColDof11 | 0.30 | 0.30 | 0.26 | 0.28 | 0.18 | 0.12 | 0.45 | 50.79 | 0.50 | 0.56 | 300 | 309 | 0.04 |
| ColDof12 | 0.42 | 0.28 | 0.31 | 0.28 | 0.23 | -0.08 | 0.39 | 54.04 | 0.42 | 0.44 | 369 | 386 | 0.07 |
| ColDof13 | 0.34 | 0.22 | 0.30 | 0.28 | 0.18 | 0.07 | 0.43 | 55.85 | 0.43 | 0.53 | 115 | 118 | 0.08 |
| ColDof14 | 0.35 | 0.29 | 0.32 | 0.30 | 0.20 | -0.03 | 0.42 | 52.24 | 0.46 | 0.48 | 171 | 176 | 0.09 |
| ColDof15 | 0.42 | 0.26 | 0.41 | 0.23 | 0.18 | -0.10 | 0.36 | 47.82 | 0.36 | 0.37 | 754 | 799 | 0.18 |
| ColDof16 | 0.37 | 0.32 | 0.22 | 0.35 | 0.20 | 0.00 | 0.44 | 48.20 | 0.52 | 0.50 | 216 | 224 | 0.09 |
| ColDof17 | 0.38 | 0.31 | 0.22 | 0.35 | 0.20 | -0.01 | 0.44 | 45.96 | 0.52 | 0.50 | 216 | 224 | 0.09 |
| ColDof18 | 0.40 | 0.33 | 0.25 | 0.19 | 0.16 | -0.04 | 0.36 | 46.07 | 0.44 | 0.47 | 265 | 285 | 0.06 |
| ColDof19 | 0.31 | 0.33 | 0.23 | 0.28 | 0.22 | 0.17 | 0.49 | 51.67 | 0.52 | 0.55 | 277 | 285 | 0.05 |
| ColDof20 | 0.44 | 0.22 | 0.43 | 0.20 | 0.17 | -0.12 | 0.34 | 51.43 | 0.31 | 0.34 | 2066 | 2132 | 0.14 |
| ColDof21 | 0.44 | 0.22 | 0.36 | 0.24 | 0.17 | -0.11 | 0.34 | 52.13 | 0.35 | 0.38 | 1667 | 1725 | 0.13 |
| ColDof22 | 0.42 | 0.23 | 0.36 | 0.25 | 0.17 | -0.09 | 0.35 | 53.44 | 0.37 | 0.39 | 1315 | 1360 | 0.13 |
| ColDof23 | 0.46 | 0.21 | 0.36 | 0.24 | 0.18 | -0.11 | 0.34 | 53.23 | 0.34 | 0.38 | 1768 | 1827 | 0.13 |
| ColDof24 | 0.45 | 0.22 | 0.36 | 0.24 | 0.18 | -0.10 | 0.35 | 55.04 | 0.35 | 0.39 | 1336 | 1380 | 0.12 |
| ColDof25 | 0.34 | 0.28 | 0.33 | 0.27 | 0.19 | 0.02 | 0.40 | 54.40 | 0.44 | 0.45 | 581 | 599 | 0.10 |
| ColDof26 | 0.43 | 0.29 | 0.29 | 0.22 | 0.19 | -0.12 | 0.36 | 57.16 | 0.41 | 0.45 | 281 | 297 | 0.10 |
| ColDof27 | 0.44 | 0.21 | 0.42 | 0.21 | 0.20 | -0.09 | 0.37 | 50.05 | 0.32 | 0.38 | 881 | 907 | 0.12 |
| ColDof28 | 0.42 | 0.27 | 0.34 | 0.22 | 0.20 | -0.02 | 0.42 | 53.90 | 0.38 | 0.44 | 548 | 566 | 0.11 |
| ColDof29 | 0.35 | 0.31 | 0.45 | 0.17 | 0.17 | -0.02 | 0.40 | 47.16 | 0.37 | 0.39 | 604 | 623 | 0.10 |
| ColDof30 | 0.42 | 0.23 | 0.47 | 0.19 | 0.19 | -0.07 | 0.38 | 47.98 | 0.31 | 0.37 | 622 | 642 | 0.10 |
| ColDof31 | 0.37 | 0.25 | 0.43 | 0.20 | 0.16 | -0.11 | 0.34 | 49.57 | 0.35 | 0.42 | 526 | 536 | 0.11 |
| ColDof32 | 0.35 | 0.26 | 0.43 | 0.20 | 0.16 | -0.11 | 0.33 | 51.58 | 0.37 | 0.42 | 523 | 534 | 0.12 |
| ColDof33 | 0.41 | 0.26 | 0.38 | 0.24 | 0.18 | -0.13 | 0.34 | 47.73 | 0.37 | 0.38 | 544 | 581 | 0.12 |
| ColDof34 | 0.34 | 0.30 | 0.32 | 0.27 | 0.21 | 0.05 | 0.43 | 53.23 | 0.46 | 0.46 | 468 | 489 | 0.12 |
| ColDof35 | 0.43 | 0.27 | 0.28 | 0.22 | 0.20 | 0.03 | 0.43 | 51.38 | 0.40 | 0.46 | 442 | 460 | 0.11 |
| ColDof36 | 0.41 | 0.29 | 0.36 | 0.20 | 0.21 | 0.00 | 0.41 | 51.70 | 0.39 | 0.40 | 628 | 658 | 0.16 |
| ColDof37 | 0.42 | 0.23 | 0.38 | 0.26 | 0.17 | -0.09 | 0.35 | 52.23 | 0.36 | 0.38 | 966 | 1006 | 0.14 |
| ColDof38 | 0.39 | 0.22 | 0.46 | 0.20 | 0.18 | -0.07 | 0.38 | 49.78 | 0.32 | 0.38 | 982 | 1006 | 0.10 |
| ColDof39 | 0.43 | 0.26 | 0.37 | 0.26 | 0.21 | -0.08 | 0.38 | 53.17 | 0.38 | 0.40 | 968 | 997 | 0.14 |
| ColDof40 | 0.45 | 0.23 | 0.37 | 0.25 | 0.19 | -0.10 | 0.37 | 52.10 | 0.36 | 0.40 | 1039 | 1073 | 0.12 |
| ColDof41 | 0.44 | 0.21 | 0.42 | 0.23 | 0.17 | -0.11 | 0.34 | 50.27 | 0.32 | 0.35 | 1114 | 1160 | 0.14 |
| ColDof42 | 0.47 | 0.24 | 0.32 | 0.22 | 0.18 | -0.12 | 0.35 | 48.10 | 0.36 | 0.40 | 693 | 724 | 0.12 |
| ColDof43 | 0.44 | 0.31 | 0.25 | 0.21 | 0.24 | 0.02 | 0.45 | 50.73 | 0.42 | 0.49 | 249 | 260 | 0.09 |
| ColDof44 | 0.29 | 0.21 | 0.25 | 0.36 | 0.13 | 0.06 | 0.39 | 52.00 | 0.50 | 0.52 | 274 | 299 | 0.07 |
| ColDof45 | 0.27 | 0.36 | 0.29 | 0.27 | 0.21 | 0.06 | 0.44 | 54.46 | 0.52 | 0.49 | 231 | 240 | 0.10 |
